# High‐Throughput, Label‐Free and Slide‐Free Histological Imaging by Computational Microscopy and Unsupervised Learning

**DOI:** 10.1002/advs.202102358

**Published:** 2021-11-07

**Authors:** Yan Zhang, Lei Kang, Ivy H. M. Wong, Weixing Dai, Xiufeng Li, Ronald C. K. Chan, Michael K. Y. Hsin, Terence T. W. Wong

**Affiliations:** ^1^ Translational and Advanced Bioimaging Laboratory Department of Chemical and Biological Engineering The Hong Kong University of Science and Technology Kowloon Hong Kong China; ^2^ Department of Anatomical and Cellular Pathology The Chinese University of Hong Kong Shatin Hong Kong China; ^3^ Department of Cardiothoracic Surgery Queen Mary Hospital Kowloon Hong Kong China

**Keywords:** computational microscopy, histology, label‐free imaging, unsupervised learning

## Abstract

Rapid and high‐resolution histological imaging with minimal tissue preparation has long been a challenging and yet captivating medical pursuit. Here, the authors propose a promising and transformative histological imaging method, termed computational high‐throughput autofluorescence microscopy by pattern illumination (CHAMP). With the assistance of computational microscopy, CHAMP enables high‐throughput and label‐free imaging of thick and unprocessed tissues with large surface irregularity at an acquisition speed of 10 mm^2^/10 s with 1.1‐µm lateral resolution. Moreover, the CHAMP image can be transformed into a virtually stained histological image (Deep‐CHAMP) through unsupervised learning within 15 s, where significant cellular features are quantitatively extracted with high accuracy. The versatility of CHAMP is experimentally demonstrated using mouse brain/kidney and human lung tissues prepared with various clinical protocols, which enables a rapid and accurate intraoperative/postoperative pathological examination without tissue processing or staining, demonstrating its great potential as an assistive imaging platform for surgeons and pathologists to provide optimal adjuvant treatment.

## Graphical abstract

A rapid, label‐free, and slide‐free histological imaging method, termed computational high‐throughput autofluorescence microscopy by pattern illumination (CHAMP) is proposed. With the assistance of computational imaging and unsupervised learning, CHAMP enables rapid and accurate pathological examination without tissue processing or staining, demonstrating great potential as an assistive imaging platform for surgeons and pathologists to provide optimal adjuvant treatment intraoperatively.

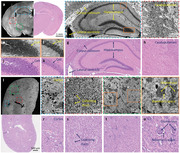

## 1 Introduction

Postoperative histological examination by pathologists remains the gold standard for surgical margin assessment (SMA), which aims to examine if there are any remaining cancer cells at the cut margin.^[^ ^1^ ^]^ However, routine pathological examination based on formalin‐fixed and paraffin‐embedded (FFPE) tissues involves a series of lengthy and laborious steps (Figure S1a, Supporting Information), causing a significant delay (ranging from hours to days) in providing accurate diagnostic reports. Although intraoperative frozen section can serve as a rapid alternative for SMA, it suffers from freezing artifacts when dealing with edematous and soft tissues, and sub‐optimal cutting for fatty tissues, affecting slide interpretation and diagnostic accuracy.^[^ ^2^ ^]^

The great demand in histopathology has inspired lots of efforts in achieving a rapid and non‐invasive diagnosis for unprocessed tissues. Some cutting‐edge microscopy techniques (Figure S2, Supporting Information) with optical sectioning capability enable slide‐free imaging of thick resection specimens, greatly simplifying the procedures associated with tissue sectioning in conventional FFPE. The scanning‐based depth‐resolved approaches, including confocal microscopy,^[^ ^3^, ^4^ ^]^ photoacoustic microscopy (PAM),^[^ ^5^, ^6^ ^]^ multiphoton microscopy (MPM),^[^ ^7^ ^]^ stimulated Raman scattering (SRS),^[^ ^8^, ^9^ ^]^ second harmonic generation (SHG),^[^ ^10^ ^]^ and their spectral multiplexing,^[^ ^11^, ^12^ ^]^ enables surface profiling of bulk tissues via 2D/3D scanning of a tightly focused laser beam. However, the imaging throughput is therefore restricted to tens of megapixels (Figure S2, Supporting Information) due to the low repetition rate of pulsed lasers and long pixel dwell time, posing a challenge to examine large specimens (e.g., human biopsies) with a centimeter‐scale surface area within a short diagnostic time frame. In contrast, wide‐field depth‐resolved techniques, including microscopy with ultraviolet surface excitation (MUSE),^[^ ^13^, ^14^ ^]^ light‐sheet microscopy,^[^ ^15^, ^16^ ^]^ and structured illumination microscopy (SIM),^[^ ^17^, ^18^, ^19^, ^20^ ^]^ are fundamentally suitable for time‐sensitive applications as their imaging throughput can reach hundreds of megapixels via parallel pixel acquisition. However, fluorescence labelling is generally required by these methods to visualize features that are analogous to FFPE histology, which is challenging to be integrated into the current clinical practice. Given this, label‐free imaging contrast is highly desirable in modern clinical settings.

The aforementioned PAM and nonlinear microscopy play an indispensable role in non‐invasive label‐free characterization of various biological structures, through either absorption‐induced thermoelastic expansion (PAM), intrinsic autofluorescence (MPM), molecular vibration (SRS), or non‐centrosymmetric orientation (SHG). The simultaneous multiplexing^[^ ^21^, ^22^ ^]^ of these methods enables cell phenotyping and classification based on the targeted biomolecules. However, the low‐throughput nature of these scanning‐based imaging modalities ultimately hinders their clinical translations. In addition, reflectance‐based imaging approaches, such as optical coherence tomography (OCT)^[^ ^23^, ^24^ ^]^ and reflectance confocal microscopy (RCM),^[^ ^25^ ^]^ pave a way for rapid label‐free inspection of human breast tissue. However, they are typically not designed at subcellular resolution.

Standard histological images are acquired at a subcellular resolution which is essential for pathological analysis. However, the inherent trade‐offs between resolution, field‐of‐view (FOV), and depth‐of‐field (DOF) fundamentally pose an impediment for rapid and high‐resolution imaging of thick tissues through a high numerical aperture (NA) objective lens. First, the image quality will be significantly degraded once the resulting shallow DOF (typically a few microns) is shorter than the optical‐sectioning thickness of the employed imaging modality, which is tunable with light‐sheet microscopy and SIM, but tissue‐dependent with MUSE (determined by the ultraviolet (UV) penetration depth (∼100 µm in human breast^[^ ^5^ ^]^ and ∼20 µm in human skin^[^ ^26^ ^]^)). In addition, the shallow DOF is unable to accommodate various surface irregularities and tissue debris presented in surgical resection specimens, leading to severe out‐of‐focus blurs which ultimately prevent high‐quality imaging of fine structures in thick specimens. Although extended DOF^[^ ^27^ ^]^ can be applied to extract in‐focus information at the tissue surface through a sequence of axially‐refocused images, the achievable throughput of the system is largely sacrificed.

Here, we propose a promising and transformative histological imaging technology, termed computational high‐throughput autofluorescence microscopy by pattern illumination (CHAMP), which enables high‐throughput and label‐free imaging of thick and unprocessed tissues with large surface irregularity at an acquisition speed of 10 mm^2^/10 s with 1.1‐µm lateral resolution. To the best of our knowledge, this is not achievable with any of the existing methods. Rich endogenous fluorophores,^[^ ^28^ ^]^ including reduced nicotinamide adenine dinucleotide (NADH), structural proteins (e.g., collagen and elastin), aromatic amino acids (e.g., tryptophan, tyrosine), and heterocyclic compounds (e.g., flavins, flavoproteins, and lipopigments), naturally form a fundamental contrast mechanism with deep‐UV excitation in CHAMP. High imaging throughput and long DOF can be achieved with the assistance of computational microscopy, making CHAMP highly suitable for intraoperative tissue assessment (e.g., SMA) where immediate feedback should be provided to surgeons for optimal adjuvant treatment. Furthermore, an unsupervised neural network is implemented to transform a CHAMP image of an unlabeled tissue into a virtually stained histological image (Deep‐CHAMP), ensuring an easy interpretation by pathologists. As thick resection tissues are inevitably deformed during the FFPE workflow (e.g., rigidity change, tissue shrinkage, tissue rupture, and slide folding), it is impractical to obtain a pixel‐to‐pixel registered label‐free CHAMP image with its corresponding histological image to form a paired training data as required by some recently reported virtual staining networks.^[^ ^29^, ^30^ ^]^ In contrast, we employ unsupervised learning based on the architecture of a cycle‐consistent generative adversarial network (CycleGAN),^[^ ^31^ ^]^ which enables image translation without paired training data, fundamentally favoring the virtual staining of CHAMP images of thick and unprocessed tissues. Diagnostic features are quantitatively extracted from Deep‐CHAMP with high accuracy. The versatility of CHAMP is experimentally demonstrated using mouse brain/kidney and human lung tissues prepared with various clinical protocols, which enables rapid and accurate intraoperative/postoperative pathological examinations without tissue processing or staining. The high‐throughput, high‐versatility, cost‐effective, and ease‐of‐use features of our CHAMP microscope hold great promise in clinical translations to revolutionize the current gold standard histopathology.

## 2 Results

### 2.1 Histological Imaging by CHAMP Microscopy

**Figure 1:**
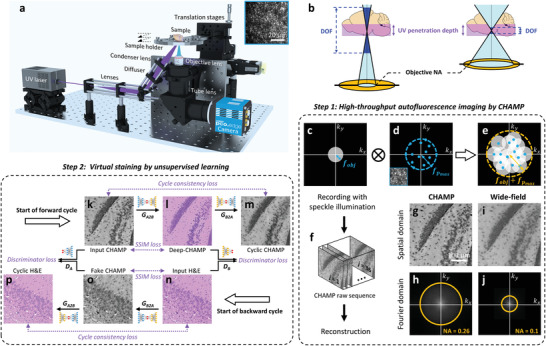
Overview of histological imaging by CHAMP. a) Schematic of the CHAMP system. The beam is expanded by a pair of lenses, and obliquely reflected onto a diffuser to generate an interference‐induced speckle pattern (inset at the top right corner), which is subsequently focused onto the bottom surface of a specimen by a condenser lens. The excited autofluorescence signal is collected by an objective lens, refocused by a tube lens, and subsequently detected by a monochromatic camera. The specimen supported by a sample holder is raster‐scanned by a 2‐axis motorized stage to generate a sequence of speckle‐illuminated diffraction‐limited autofluorescence images. b) Illustration of the relationship between DOF, objective NA, UV penetration depth (i.e., UV optical‐sectioning thickness), and tissue surface irregularity. c) The aperture of the diffraction‐limited imaging system with a cut‐off frequency of *f_obj_*. d) The spectrum of the speckle pattern with a maximum frequency of $f_{p_{max}}$. e) The synthetic aperture (cut‐off frequency = $f_{obj}+f_{p_{max}}$) through intensity modulation by speckle illumination. f) The captured raw image sequence for CHAMP reconstruction. g,h) A reconstructed resolution‐enhanced image (i.e., a CHAMP image) and its corresponding spectrum in Fourier domain, respectively. i,j) Diffraction‐limited wide‐field image captured with uniform illumination and its corresponding spectrum in the Fourier domain, respectively. k) Input CHAMP image. l) Virtually stained Deep‐CHAMP image. m) Generated cyclic CHAMP image. n) Input H&E‐stained image. o) Generated fake CHAMP image. p) Generated cyclic H&E‐stained image.

Our CHAMP microscope is configured in a reflection mode (**Figure** **1**a and Video S1, Supporting Information), which can accommodate tissues of any size and thickness without physically interfering with the illumination and collection optics. Deep‐UV laser at 266 nm, which presents a significant difference in the quantum yields between nucleotides^[^ ^32^ ^]^ and other endogenous fluorophores, is used for illumination in our CHAMP system to maximize the negative contrast of cell nuclei (Figure S3, Supporting Information). Oblique illumination circumvents the use of UV‐transmitting optics and fluorescence filters because the backscattered UV light is naturally blocked by the glass objective and tube lens which are spectrally opaque at 266 nm. A constant speckle pattern (inset of Figure 1a), which is generated by a diffuser and featured a grain size smaller than the point spread function (PSF) of the detection optics, is projected onto the bottom surface of the specimen for pattern illumination. A long DOF (Figure 1b) enabled by the implementation of a low‐NA objective lens not only matches the optical‐sectioning thickness provided by UV surface excitation, but also accommodates different levels of tissue surface irregularities. With intensity modulation, the aperture of the diffraction‐limited system (Figure 1c) is convolved with the spectrum of speckle pattern which contains various frequency components (Figure 1d), and is consequently 2D translated in the Fourier domain, enabling the synthesis of an extended system passband (Figure 1e). This allows high spatial frequency (i.e., high‐resolution features) to be encoded into the low‐NA imaging system, thus bypassing the resolution limit governed by the low‐NA objective lens equipped in the CHAMP microscope. The sample is raster‐scanned to generate a sequence of speckle‐illuminated diffraction‐limited images (Figure 1f), which are subsequently demodulated to reconstruct a resolution‐enhanced image (Figure 1g and Video S1, Supporting Information) (termed CHAMP image hereafter). CHAMP imaging features 2.6× resolution improvement (see Methods, Figure 1h,j) compared with conventional wide‐field microscopy with uniform illumination (Figure 1i). The reconstructed CHAMP image is subsequently transformed into a virtually stained histological image (termed Deep‐CHAMP image hereafter) through a CycleGAN‐based neural network, which is composed of four deep neural networks, including two generators (*G*_A2B_, *G*_B2A_) and two discriminators (*D*_A_, *D*_B_). The generator *G_A2B_* learns to transform grayscale images to color images, while the generator *G_B2A_* learns to transform color images to grayscale images. A sequence of unpaired CHAMP images and hematoxylin and eosin (H&E) stained images are fed to the neural network to undergo a forward training cycle (Figure 1k–m) and a backward training cycle (Figure 1n–p). The discriminator *D_A_* aims to distinguish real input CHAMP images (Figure 1k) from fake CHAMP images (Figure 1o) produced by the generator *G*_B2A_. Meanwhile, the discriminator *D*_B_ aims to distinguish real input H&E‐stained images (Figure 1n) from virtually stained Deep‐CHAMP images (Figure 1l) produced by the generator *G*_A2B_. Once the generator *G*_A2B_ can produce Deep‐CHAMP images that the discriminator *D*_B_ cannot distinguish from the input H&E‐stained images, the transformation from CHAMP to Deep‐CHAMP is well learned by the generator *G*_A2B_. This iterative process is also applicable to the generator *G*_B2A_ and the discriminator *D*_A_.

### 2.2 CHAMP and Histological Imaging of Thin Mouse Brain/Kidney Tissue Slices

**Figure 2:**
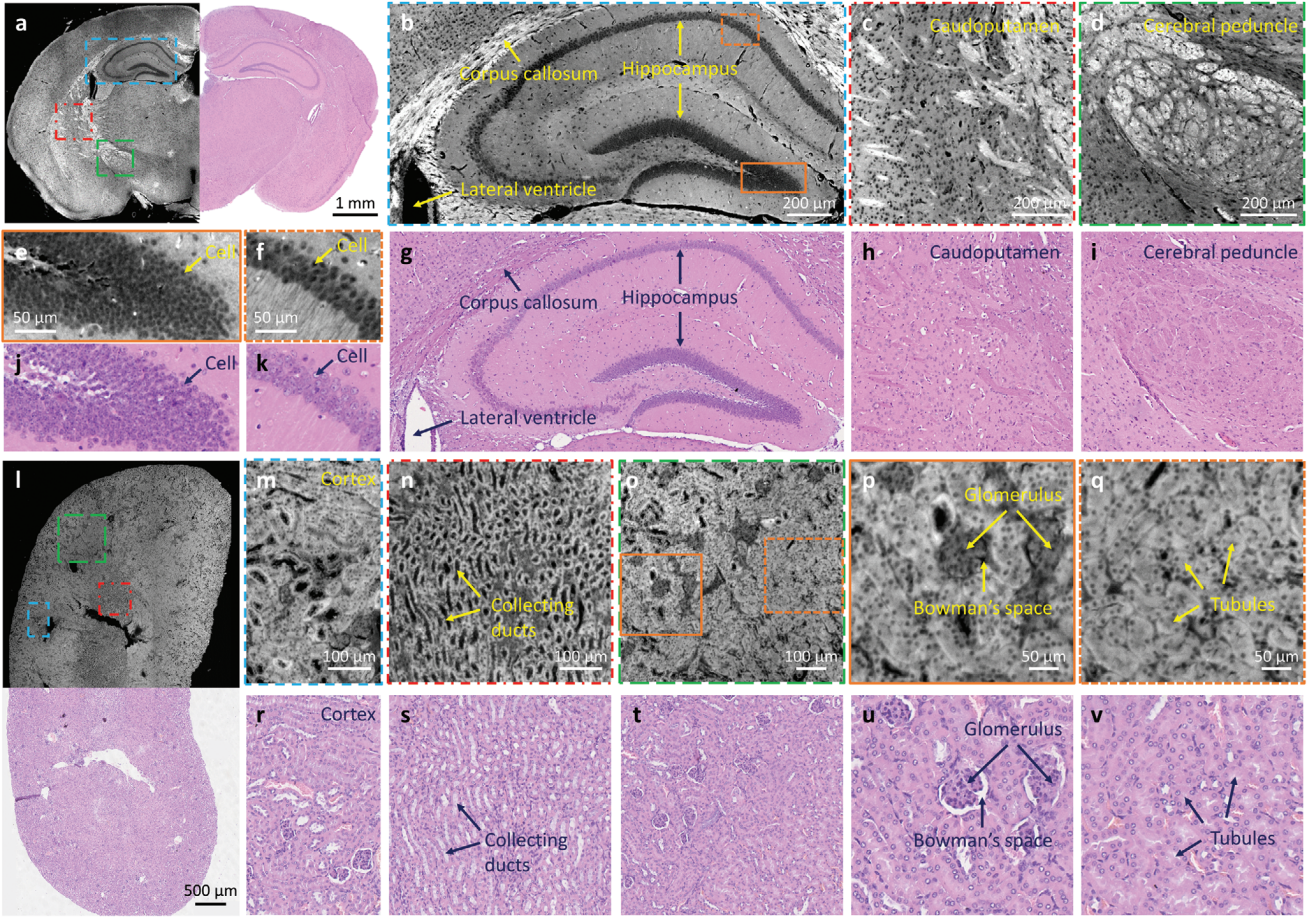
CHAMP and histological imaging of thin mouse brain/kidney tissue slices. a) Combined CHAMP and H&E‐stained mosaic image of a mouse brain. b–d) Zoomed‐in CHAMP images of blue, red, and green dashed regions in (a), respectively. e,f) Zoomed‐in CHAMP images of orange solid and dashed regions in (b), respectively. g–k) The corresponding H&E‐stained images. l) Combined CHAMP and H&E‐stained mosaic image of a mouse kidney. m–o) Zoomed‐in CHAMP images of blue, red, and green dashed regions in (l), respectively. p,q) Zoomed‐in CHAMP images of orange solid and dashed regions in (o), respectively. r–v) The corresponding H&E‐stained images.

FFPE thin slices of mouse brain/kidney tissues are imaged to validate the performance of CHAMP initially (**Figure** **2**). The microtome‐sectioned thin slices (with thickness  ∼7 µm) are deparaffinized before CHAMP imaging. Cell nuclei distributed at the cerebral cortex and brain stem are clearly revealed with a negative contrast in CHAMP images. With a measured resolution of 1.1 µm (see Methods), the densely packed cell nuclei in the hippocampus (Figure 2b, and zoomed‐in CHAMP images (Figure 2e,f) of orange solid and dashed regions in Figure 2b) can be resolved individually. Other anatomical structures, including lateral ventricle and corpus callosum (Figure 2b), caudoputamen (Figure 2c), and cerebral peduncle (Figure 2d) are also well recognized. After CHAMP imaging, the slice is histologically stained by H&E, and imaged with a bright‐field microscope to obtain the corresponding histological images (Figure 2g–k). The cerebral peduncle, which is poorly visualized in the H&E‐stained image (Figure 2i), can be clearly identified in CHAMP (Figure 2d). Multiple similarities are revealed in CHAMP and H&E‐stained images, despite that the nucleoli are less visible in CHAMP. Pearson correlation coefficient of 0.9 is calculated from Figure 2b,g, validating the feasibility of using tissue's autofluorescence as an intrinsic contrast mechanism for label‐free characterization of biological structures. Similarly, CHAMP provides well‐characterized structures of a mouse kidney (Figure 2l–q), including cortex (Figure 2m), collecting ducts (Figure 2n), glomerulus and Bowman's space (Figure 2p), and renal tubules (Figure 2q). Their corresponding H&E‐stained images are shown in Figure 2r–v. A reduced correlation coefficient of 0.7 is calculated from Figure 2p,u, which is due to the locally deformed Bowman's space during the subsequent H&E staining after CHAMP imaging.

### 2.3 CHAMP Imaging of Thick and Unprocessed Mouse Brain/Kidney Tissues

**Figure 3:**
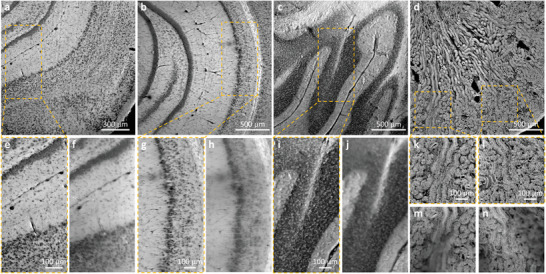
CHAMP imaging of thick and unprocessed mouse brain/kidney tissues. a–c) CHAMP images of fixed and unprocessed mouse brain tissues hand‐cut at different coronal planes with thickness ∼ 5 mm. d) CHAMP image of a fixed and unprocessed mouse kidney tissue sectioned with thickness ∼200 µm. e,g,i,k,l) Zoomed‐in CHAMP images of orange dashed regions in (a–d). f,h,j,m,n) The corresponding wide‐field images captured with a 0.3‐NA imaging objective with uniform illumination.

To showcase the slide‐free and label‐free imaging capability of CHAMP as well as its superiority in thick tissue imaging, formalin‐fixed and unprocessed thick mouse brain/kidney tissues are imaged (**Figure** **3**). The mouse brain tissues are hand‐cut at different coronal planes with thickness  ∼5 mm, while the mouse kidney tissue is vibratome‐sectioned with a thickness  ∼200 µm. As mentioned above, resolution‐enhanced and all‐in‐focus (due to the long DOF) CHAMP images (Figure 3a–d) eliminate any image blur that potentially originated from mismatched DOF and UV penetration depth or non‐flattened tissue surface. Zoomed‐in CHAMP images of cell nuclei in the hippocampus (Figure 3e,g) and lobules (Figure 3i) far outperform the corresponding wide‐field images which are directly captured with a 0.3‐NA imaging objective (Figure 3f,h,j). The out‐of‐focus blurs presented in kidney tubules (Figure 3m,n) are eliminated in CHAMP images (Figure 3k,l) such that individual cells are clearly observed in the entire FOV.

### 2.4 CHAMP and Deep‐CHAMP Imaging of Tissues Treated with Various Clinical Protocols

**Figure 4:**
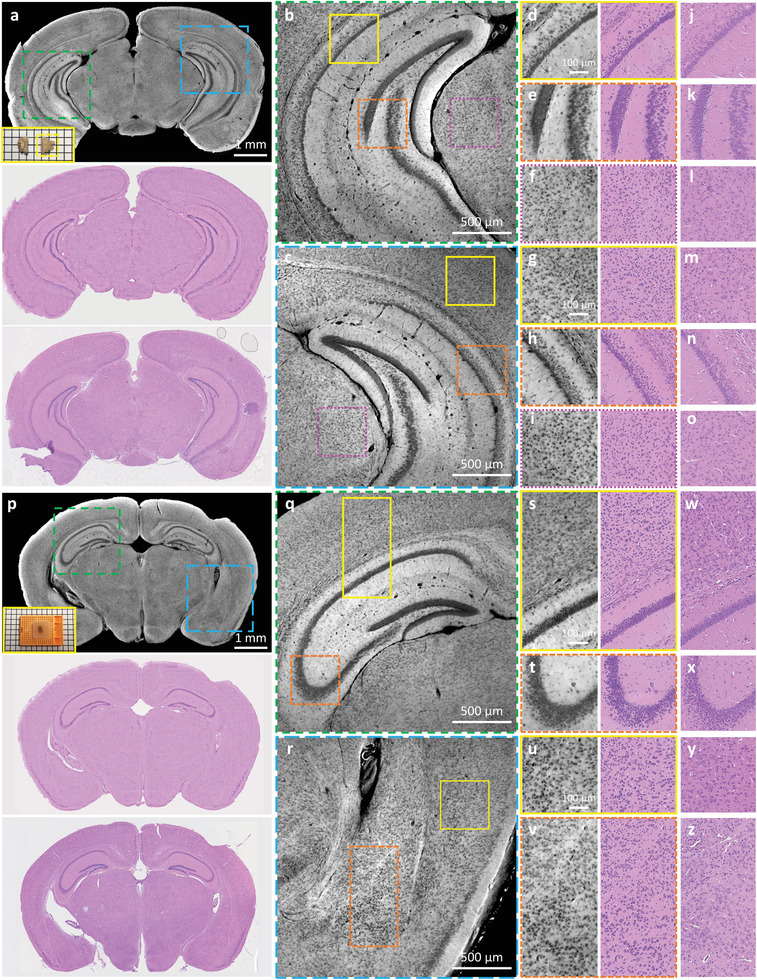
CHAMP and Deep‐CHAMP validation with formalin‐fixed thick mouse brains. a) CHAMP (top) and Deep‐CHAMP (middle) images of a fixed and unprocessed mouse brain, inset at the bottom left of the CHAMP image shows the photograph of the specimen (the yellow dashed box shows the mouse brain that is imaged). The corresponding H&E‐stained thin slice image (bottom). b,c) Zoomed‐in CHAMP images of green and blue dashed regions in (a), respectively. d–f,g–i) Zoomed‐in CHAMP and Deep‐CHAMP images of yellow solid, orange dashed, and magenta dashed regions in (b) and (c), respectively. j–o) The corresponding H&E‐stained images. p) CHAMP (top) and Deep‐CHAMP (middle) images of a fixed and paraffin‐embedded mouse brain, inset at the bottom left of the CHAMP image shows the photograph of the specimen. The corresponding H&E‐stained thin slice image (bottom). q,r) Zoomed‐in CHAMP images of green and blue dashed regions in (p), respectively. s–v) Zoomed‐in CHAMP and Deep‐CHAMP images of yellow solid and orange dashed regions in (q) and (r), respectively. w–z) The corresponding H&E‐stained images.

CHAMP and Deep‐CHAMP imaging is experimentally validated with mouse brain/kidney tissues and human lung cancer tissues which are treated with various clinical protocols, e.g., microtome‐sectioned thin tissue slice (Figure S4, S5 and Video S2, Supporting Information), formalin‐fixed thick tissues (**Figure** **4** and Video S3, Supporting Information, **Figure** **5**, Figure S6 and Video S4, Supporting Information), as well as freshly excised tissues (**Figure** **6**, Figure S7, Supporting Information). We trained two neural networks to separately handle the virtual staining of fixed and fresh mouse brains due to the significant difference in CHAMP images. In addition, we found the overall trend for the CycleGAN is that it converts brighter regions to white background, and darker regions to purple nuclei. Therefore, dark features in CHAMP (e.g., interstitial spaces, ventricles, and vessels) can be incorrectly color mapped to purple and mixed with cells. To alleviate this issue, the CHAMP image is segmented by a pre‐trained classifier to separate cell nuclei from features that demonstrate similar brightness. After that, the segmented CHAMP image is cropped and fed into the network to output a virtually stained Deep‐CHAMP image.

**Figure 5:**
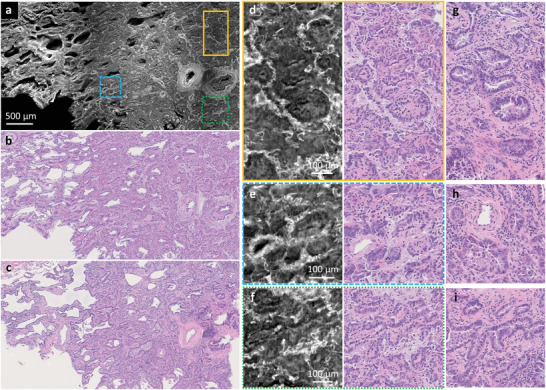
CHAMP and Deep‐CHAMP validation with a formalin‐fixed and unprocessed human lung tissue. a,b) CHAMP and Deep‐CHAMP images of a formalin‐fixed and unprocessed human lung tissue with adenocarcinoma, respectively. c) The corresponding H&E‐stained thin slice image. d–f) Zoomed‐in CHAMP and Deep‐CHAMP images of orange solid, blue dashed, and green dashed regions in (a), respectively. g–i) The corresponding H&E‐stained images.

**Figure 6:**
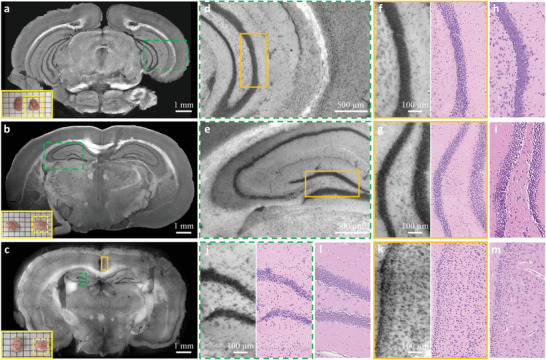
CHAMP and Deep‐CHAMP validation with freshly excised mouse brains. a–c) CHAMP images of freshly excised mouse brains, inset at the bottom left of each figure shows the photograph of the specimen (the yellow dashed box indicates the mouse brain that is imaged). d,e) Zoomed‐in CHAMP images of green dashed regions in (a) and (b), respectively. f,g) Zoomed‐in CHAMP and Deep‐CHAMP images of orange regions in (d) and (e), respectively. h,i) The corresponding H&E‐stained images. j,k) Zoomed‐in CHAMP and Deep‐CHAMP images of green dashed and orange solid regions in (c), respectively. l,m) The corresponding H&E‐stained images.

In Figure 4, the freshly excised mouse brains are fixed in formalin for 24 h to prevent tissue degradation, after which the specimens are manually sectioned without any further processing (Figure 4a), or processed and paraffin‐embedded as a block tissue (Figure 4p). The specimens are imaged by CHAMP and virtually stained to generate the corresponding Deep‐CHAMP images (Figure 4d–i,s–v), and are subsequently processed by a standard histological procedure to obtain the H&E‐stained images for comparison (Figure 4j–o,w–z). Note that the microtome‐sectioned FFPE thin slice is not able to exactly replicate the surface imaged by CHAMP due to the tissue deformation and the difference in imaging thickness. Despite this difference, the structural features are still remarkably similar.

As an initial clinical value validation of CHAMP and Deep‐CHAMP, a thin human lung cancer tissue with large cell carcinoma is first imaged (Figure S5). Both CHAMP, Deep‐CHAMP, and H&E‐stained images outline a clear interface between the normal and tumor regions (Figure S5a–c, Supporting Information). The alveoli structures with air spaces are shown in the Deep‐CHAMP image of the normal lung tissue region (Figure S5e, Supporting Information). Although the alveoli septa appear slightly thicker than that of the H&E‐stained image (Figure S5f, Supporting Information), it still falls well within the spectrum of normal morphology, and the airway looks essentially identical. For the tumor region, large cancer cells can be easily observed in all three sets of images (Figure S5g–i, Supporting Information).

To further demonstrate the powerfulness of CHAMP and Deep‐CHAMP imaging in an intraoperative setting, a formalin‐fixed and thick human lung adenocarcinoma tissue is imaged (Figure 5a,b) with the corresponding H&E‐stained image as a reference (Figure 5c). The CHAMP and Deep‐CHAMP images (Figure 5d–f) show the loss of alveoli, replaced by cancer tissue arranged in mostly acini structure separated by desmoplastic stroma. The acini structures are lined by cancerous pneumocytes showing nuclear atypia, which is a sign of cellular dysregulation and is often related to cancer development. The degree of nuclear atypia visualized by Deep‐CHAMP is similar to that in the H&E‐stained images (Figure 5g–i). Overall, the destruction of the architecture and nuclear atypia depicted in Deep‐CHAMP are sufficient to reach a diagnosis of adenocarcinoma by a pathologist with minimal difficulty.

In Figure 6, freshly excised mouse brains are rinsed in phosphate‐buffered saline to remove adhesive blood on the cut surface, and blotted up before imaging. The specimens are imaged by CHAMP and virtually stained to generate the corresponding Deep‐CHAMP images (Figure 5f,g,j,k). After that, the specimens are processed following the standard procedure to obtain the H&E‐stained images (Figure 5h,i,l,m). Despite the tissue deformation and shrinkage of fresh brains (the percentage of shrinkage is ∼40% in our experiments), the histological features are still considerably similar. It should be noted that cell nuclei located at heavily myelinated regions are partially obscured due to the strong scattering in fresh brain tissues.

In addition to fresh mouse brain, CHAMP and Deep‐CHAMP imaging are also applied to a fresh mouse kidney (Figure S7, Supporting Information). The densely packed cell nuclei along kidney tubules are well‐identified in CHAMP (Figure S7b–e, Supporting Information). These CHAMP images are first fed to the virtual staining network trained for fresh mouse brain to obtain “brain‐style” Deep‐CHAMP images (Figure S7f–i, Supporting Information), which are subsequently input to another unsupervised network trained for style transformation (see Methods), to generate “kidney‐style” Deep‐CHAMP images (Figure S7j–m, Supporting Information). This bridge network allows style transformation among different types of tissues without the need for retraining on specific tissue, demonstrating great simplicity and flexibility of the unsupervised neural networks. Note that intricate renal tubules and vessels in a fresh kidney pose a great challenge for feature segmentation, which consequently leads to some staining artifacts in the generated Deep‐CHAMP images (indicated by the arrows in Figure S7, Supporting Information).

**Figure 7:**
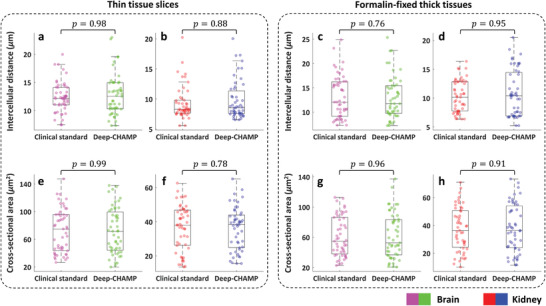
Distributions of nuclear features extracted from Deep‐CHAMP and clinical standard images. a–d) Intercellular distances extracted from thin mouse brain/kidney slices (a,b) and formalin‐fixed thick mouse brain/kidney tissues (c,d), respectively. e–h) Cross‐sectional areas extracted from thin mouse brain/kidney slices (e,f) and formalin‐fixed thick mouse brain/kidney tissues (g,h), respectively. Wilcoxon rank‐sum testing is carried out across groups with *n* = 50 for each distribution. The significance is defined as p* ≤ 0.05 in all cases.

Diagnostic features, such as cross‐sectional area and intercellular distance of the cell nuclei, play an important role in tissue phenotyping and histologic tumor grading.^[^ ^33^, ^34^ ^]^ These features can be quantitatively extracted from Deep‐CHAMP with high accuracy. As shown in **Figure** **7**, nuclear features are derived from FFPE thin mouse brain/kidney slices (Figures S4g,m and S4t,x, Supporting Information), and formalin‐fixed and unprocessed thick mouse brain/kidney tissues (Figure 4i,o and Figure S6f,j, Supporting Information). Wilcoxon rank‐sum testing is applied to evaluate the difference in nuclear features extracted from Deep‐CHAMP and gold standard H&E‐stained images. Our results show that the distributions of cross‐sectional area and intercellular distance extracted from Deep‐CHAMP agree fairly well with the H&E‐stained images in both thin and thick tissue specimens. Although cell counting may be slightly affected by feature segmentation, the distributions of nuclear features still support the accuracy of the information that can be extracted from the Deep‐CHAMP. These results are highly encouraging and suggest that CHAMP/Deep‐CHAMP can be potentially translated into the current histopathological practice to alleviate the workload involved in the frozen section or FFPE tissue preparation (Figure S1b, Supporting Information).

## 3 Discussion

CHAMP is a promising and transformative histological imaging technology that enables rapid, label‐free, and high‐resolution imaging of thick and unprocessed tissues, holding great promise to streamline the standard‐of‐care histopathology. However, there are still challenges ahead as CHAMP and H&E‐stained images exhibit some deviations. First, the nucleoli structures are better visualized in H&E‐stained images than that in CHAMP under the same magnification (e.g., Figures 2f,k, and 4v,z). This is likely because the fluorescence property of nucleoli in the detected spectral range is not chemically identical to H&E histological stains. Second, the densely packed cell nuclei in the hippocampus are less distinguishable in CHAMP compared with the clinical standard images in fresh tissue (e.g., Figure 6f,h). This may be attributed to the difference in the imaged thickness (tens of micrometers in CHAMP versus 7 µm in H&E‐stained images). Third, fiber tracts are better visualized in CHAMP than that in H&E‐stained images (e.g., Figures 2b–d,g–i, and 5d–f, g–i). This is possibly due to the proteins in these fibrous structures present a high quantum yield under deep‐UV excitation while eosin exhibits a similar affinity as with cytoplasm. Fourth, pyramidal cell nuclei in the hippocampus are better identified in the H&E‐stained images than that in Deep‐CHAMP (Figure 6f–i). This is a limitation of the current unsupervised network, which tends to learn better from image brightness than structures even though the structural similarity loss is implemented in the network (see Section 4). Thus, Deep‐CHAMP is less effective to preserve fine structures with low image contrast. Furthermore, the pyramidal cell nuclei are with less population, causing insufficient data for training. Therefore, we believe that integrating a large training dataset or imposing weak/semi‐supervision to the current network could be a solution for this problem. In addition, equipping CHAMP with higher optical‐sectioning capability is feasible to mitigate the thickness‐induced deviations between Deep‐CHAMP and H&E‐stained images. The axial super‐resolution contents can be provided by 3D structured illumination approaches,^[^ ^35^, ^36^ ^]^ which could potentially facilitate a more comprehensive specimen analysis.

Although an exogenous contrast agent is not required for CHAMP imaging, significant variations in autofluorescence intensity can still be observed in CHAMP as the excitation light is scattered/absorbed differently among various tissue types and functional areas (e.g., white matter and gray matter of the brain).^[^ ^37^ ^]^ The fluorescence properties of intrinsic fluorophores, such as excitation/emission maximum and quantum yield, are highly related to the biochemical environment and disease status. For instance, tissue fixation by using formaldehyde or glutaraldehyde leads to a significant increase in autofluorescence intensity, especially in the kidney and spleen. However, this has little effect on the Deep‐CHAMP images as the fluorescence intensity increases for the entire image, and hence, the relative variation is minimal. For disease status, we found that the membrane of alveolar macrophages in cancerous human lung tissues is highly fluorescent with deep‐UV excitation (indicated by arrows in Figure S5d). Still, the nuclei are dark with high consistency, which is essential for the high‐quality virtual staining (Figure S5e,f, Supporting Information). In addition, a transitory decrease in autofluorescence, which is related to UV‐induced photo‐oxidation,^[^ ^38^ ^]^ can occur at the beginning of UV radiation. With continuous exposure, the intensity is progressively increased, and the resulting homogenously distributed autofluorescence adversely degrades the negative contrast that can be observed in the CHAMP images. This could potentially be a problem for Deep‐CHAMP as the current virtual staining network achieves color transformation primarily based on the image brightness. We believe that this issue can be addressed with neural network development. Situations are more complicated in fresh tissues, where different levels of local hemorrhage can occur during excision with biopsy forceps, and the consequent attenuation of radiation due to the absorption by non‐fluorescent chromophores like hemoglobin^[^ ^39^ ^]^ should also be taken into account. The proposed method is at an early stage of development. The influence of autofluorescence variability on the diagnosis of various organs and diseases still requires large‐scale clinical studies. The reliability of CHAMP/Deep‐CHAMP can be further improved by (i) the assistance of autofluorescence spectroscopy, as metabolic enzymes such as NADH and FAD can effectively serve as a biomarker to differentiate normal tissues from malignant lesions,^[^ ^28^ ^]^ and by (ii) the development of deep‐learning algorithms.

CHAMP can potentially reach higher imaging speed to further shorten the diagnostic timeframe for more time‐sensitive applications. Currently, the imaging speed is limited by the exposure time of each speckle‐illuminated image, which is nearly 280 ms with an illumination power of 2 mW to maintain a good signal‐to‐noise ratio (SNR). The acquisition speed can be further accelerated by increasing excitation power. Another limiting factor is the number of acquisitions that are required for super‐resolution reconstruction. The minimum number of acquisitions is related to the sparsity of the imaged features. According to our experiments, a sufficiently large scanning range (greater than twice the length of the low‐NA diffraction‐limited spot size) and fine scanning steps (smaller than the targeted resolution) can reduce distortions in the reconstruction. 2.6× resolution improvement can be obtained through at least 36 speckle‐illuminated (average grain size ∼ 1.3 µm) autofluorescence images without obvious degradation in the reconstructed CHAMP image. The system can be further optimized to balance the achievable resolution and acquisition speed for a specific application. Note that the computational efficiency may be a dominant impediment for CHAMP imaging, which takes ∼50 s/10 mm^2^ due to the use of an iterative reconstruction framework that involves a massive number of Fourier transformations. This issue can be potentially addressed by the implementation of powerful computational resources or the introduction of a deep‐learning approach,^[^ ^40^ ^]^ which allows super‐resolution reconstruction with a reduced number of acquisitions under low light conditions. This is expected to dramatically speed up structured illumination approaches and release the computational burden of CHAMP imaging.

It should be admitted that the CycleGAN‐based network faces difficulty to differentiate features that demonstrate similar brightness (e.g., cell nuclei with interstitial spaces, ventricles, and vessels), which can lead to some staining artifacts (as indicated by the arrows in Figures S4 and S7, Supporting Information). We believe that integrating weakly‐/semi‐supervised data or introducing a saliency constraint^[^ ^41^ ^]^ would help to address this problem, further improving the accuracy of Deep‐CHAMP images. Virtual staining through unsupervised learning should be systematically investigated in the future to enable a faithful conversion, which, however, is beyond the scope of this study.

In summary, we propose a revolutionary and transformative histological imaging technology that enables rapid, label‐free, and high‐resolution imaging of thick and unprocessed tissues with large surface irregularity. The versatility of CHAMP is experimentally demonstrated, which enables rapid and accurate pathological examination without tissue processing or staining, demonstrating great potential as an assistive imaging platform for surgeons and pathologists to provide optimal adjuvant treatment intraoperatively. To show the diagnostic reliability of CHAMP/Deep‐CHAMP, large‐scale clinical trials should be carried out as follow‐up work. Moreover, computer‐aided diagnoses could be incorporated with CHAMP/Deep‐CHAMP to further improve the efficiency of the current clinical workflow.

## 4 Experimental Section

### Collection of Biological Tissues

The animal organs were extracted from C57BL/6 mice. For fresh animal tissues (Figure 6 and Figure S7, Supporting Information), the brain/kidney were harvested immediately after the mice were sacrificed and rinsed in phosphate‐buffered saline for a few seconds, and then blotted up with laboratory tissue for CHAMP imaging. To prepare fixed and unprocessed tissues (Figures 3, 4, 5, and Figure S6, Supporting Information), the freshly excised tissues were fixed in 4% neutral‐buffered formalin at room temperature for 24 h, and manually sectioned with ∼5‐mm thickness or sectioned by a vibratome (VF‐700‐0Z, Precisionary Instruments Inc.) with ∼200‐µm thickness. To prepare paraffin‐embedded tissue (Figure 4p), the formalin‐fixed tissues were processed with dehydration, clearing, and infiltration by a tissue processor (Revos, ThermoFisher Scientific Inc.) for 12 h, and paraffin‐embedded as a block specimen. To prepare thin tissue slices (Figure 2 and Figures S4,S5, Supporting Information), the paraffin‐embedded block tissues were sectioned at the surface with ∼7‐µm thickness by a microtome (RM2235, Leica Microsystems Inc.). The thin tissue slices were stained by H&E, and subsequently imaged by a digital slide scanner (NanoZoomer‐SQ, Hamamatsu Photonics K.K.) to generate the histological images. Human lung cancer tissues were obtained from lung cancer patients who underwent curative lung cancer surgery at the Queen Mary Hospital. Following lung lobectomy, the lung cancer tissues were cut with a scalpel from the resected lobe, subsequently fixed in formalin, and transported to the lab for imaging. After CHAMP imaging, the tissues were processed by the standard histological procedure to obtain the H&E‐stained images. All animal experiments were carried out in conformity with a laboratory animal protocol approved by the Health, Safety and Environment Office (HSEO) of Hong Kong University of Science and Technology (HKUST) (licence number: AH18038), whereas all human experiments were carried out in conformity with a clinical research ethics review approved by the Institutional Review Board of the University of Hong Kong/ Hospital Authority Hong Kong West Cluster (HKU/HA HKW) (reference number: UW 20–335), and informed consent was obtained from all lung cancer tissue donors.

### Reflection‐Mode CHAMP System

As shown in Figure 1a, a nanosecond UV pulsed laser is used as the excitation source (266 nm wavelength, WEDGE HF 266 nm, Bright Solutions Srl.), which is spectrally filtered by a bandpass filter (FF01‐300/SP‐25, Semrock Inc.) and expanded by a pair of lenses (LA4647‐UV and LA4874‐UV, Thorlabs Inc.). After that, the expanded beam is obliquely reflected by a UV mirror (PF10‐03‐F01, Thorlabs Inc.) and projected onto a diffuser (DGUV10‐600, Thorlabs Inc.) to generate a constant speckle pattern, which is subsequently focused onto the bottom surface of a specimen by a condenser lens (LA4148‐UV, Thorlabs Inc.) with an illumination power of 2 mW. The excited autofluorescence signal is detected by an inverted microscopy system which consists of a plan achromat infinity‐corrected objective lens (RMS4X, NA = 0.1, Thorlabs Inc.) and an infinity‐corrected tube lens (TTL180‐A, Thorlabs Inc.), and finally imaged by a monochrome scientific complementary metal‐oxide‐semiconductor (sCMOS) camera (PCO edge 4.2, 2048 × 2048 pixels, 6.5‐µm pixel pitch, PCO Inc.). In this experiment, the specimen was 2D raster‐scanned by a 2‐axis motorized stage (L‐509.20SD00, PI miCos GmbH) with a scanning interval of 1 µm. A sequence of speckle‐illuminated diffraction‐limited autofluorescence images were recorded by the sCMOS camera which was synchronized with the motor scanning via our lab‐designed LabVIEW software (National Instruments Corp.) and triggering circuits. 36 speckle‐illuminated raw images were generally required to generate a CHAMP image in this study. The data acquisition time for each raw image was set to 280 ms (250‐ms camera integration time plus 30‐ms stage settling time) to balance between the image SNR and acquisition speed, which can reach 10 mm^2^/10 s under this setting.

### Super‐Resolution Reconstruction Framework

Extended DOF enabled by the implementation of a low‐NA imaging objective in the CHAMP microscope not only matched the optical‐sectioning thickness provided by UV surface excitation, but also accommodated different levels of tissue surface irregularities. To bypass the resolution limit set by the low‐NA objective and maximize the achievable imaging throughput, structured illumination with a constant speckle pattern was implemented. Undetectable high‐frequency information can be multiplexed into the low‐NA imaging system through intensity modulation, where the fluorescent specimen was illuminated with non‐uniform intensity‐varied patterns, including sinusoidal stripe,^[^ ^42^, ^43^ ^]^ multifocal spot,^[^ ^44^, ^45^ ^]^ or random speckle patterns.^[^ ^46^, ^47^, ^48^, ^49^, ^50^, ^51^ ^]^ The processes of intensity modulation with uniform illumination, linear/nonlinear sinusoidal stripe, and random speckle pattern were respectively demonstrated in Figure S8, Supporting Information. The highest achievable resolution through structured illumination was determined by the reciprocal of Fourier space bandwidth, which is given by *λ*/2(NA_*obj*_ + NA_*illu*_), where *λ* was the fluorescence emission wavelength, and NA_*obj*_ and NA_*illu*_ were the numerical apertures of the detection objective lens and illumination pattern, respectively. The resolution improvement through conventional SIM with epi‐fluorescence configuration was restricted to 2× as the illumination NA was also restricted by the detection objective (Figure S8e–h, Supporting Information). The adoption of high‐frequency sinusoidal harmonics generated by nonlinear fluorescence response allowed reaching beyond 2× resolution enhancement^[^ ^52^, ^53^ ^]^ (Figure S8i–l, Supporting Information). However, the photodamage and photobleaching associated with high‐power excitation will hinder its biomedical applications. Recent studies showed that 4× resolution improvement can be achieved through an off‐axis projection of a set of frequency‐multiplexed sinusoidal patterns.^[^ ^54^ ^]^ However, the system complexity was inevitability increased, and the transmission‐based configuration restricts its application only to thin samples.

For simplicity and flexibility, oblique illumination with a constant speckle pattern, which featured a grain size smaller than the PSF of the detection optics, was implemented in CHAMP to obtain beyond 2× resolution enhancement. In addition, with the assistance of UV surface excitation, this method is applicable to unprocessed and unlabeled tissues with any thickness, which is not achievable with the existing super‐resolution SIM systems. Unlike SIM with sinusoidal illumination, where super‐resolution demodulation can be achieved through a one‐step analytic inversion with a few raw images, SIM with speckle illumination (e.g., CHAMP, Figure S8m–p) fails to establish a direct inversion relationship, thus requiring more redundant acquisitions to isotropically fill the Fourier space. Note that even with prior information based on speckle statistics^[^ ^49^, ^55^, ^56^ ^]^ and sample sparsity^[^ ^50^, ^57^ ^]^, multifold resolution gain was not experimentally achievable with fully‐randomized speckle patterns due to the ill‐posed nature in this situation, that is, *N* intensity measurements were captured with *N*+1 unknown variables to be solved (*N* illumination patterns plus one sample distribution). To address this issue, illuminating with a constant speckle pattern that is translated between measurements, as opposed to randomly changing speckle patterns, was utilized in this report.^[^ ^46^, ^51^, ^58^ ^]^

The CHAMP reconstruction framework is based on a momentum‐assisted regularized ptychographic iterative engine,^[^ ^59^ ^]^ which is a well‐developed inversion solver with significantly improved robustness that enables rapid convergence to a lower error (10 iterations are generally sufficient in the experiments). The flowchart of the reconstruction algorithm is shown in Table S1, Supporting Information. Before reconstruction, the raw images were flattened to correct illuminance non‐uniformity. Then, the scanning trajectory (*x_j_*,*y_j_*) of the specimen was pre‐estimated by cross‐correlation of the captured raw images.^[^ ^60^ ^]^ Note that the sampling rate is a prerequisite for digital image reconstruction. Undersampling issue, which occured in the CHAMP system as the sampling pixel size is larger than half of the PSF size of the low‐NA detection optics, will lead to pixel aliasing and consequently generate artifacts in the reconstruction. To tackle this issue, a sub‐sampled method^[^ ^61^ ^]^ was introduced. The algorithm was run on a workstation with a Core i9‐10980XE CPU @ 4.8GHz and 8×32GB RAM, and 4 NVIDIA GEFORCE RTX 3090 GPUs, which takes ∼50 s/10 mm^2^ for computation.

Note that the resolution improvement is theoretically infinite, which, however, will be experimentally restricted by the speckle contrast on the specimen. In principle, a condenser lens with higher illumination NA enables higher achievable resolution at the expense of more acquisitions. However, the resulting highly compressed speckle pattern not only causes a vignetting effect which darkens the corners of the captured autofluorescence images, but also degrades the speckle contrast due to the natural decay governed by the incoherent optical transfer function. Therefore, the system can be optimized to balance the tradeoffs between target resolution, acquisition speed, and computational efficiency for various applications. In this work, 2.6× resolution gain was achieved via 36 speckle‐illuminated (average grain size  ∼1.3 µm) diffraction‐limited images that were raster‐scanned with 1‐µm scanning interval. CHAMP enables rapid and label‐free imaging of thick and unprocessed tissues with large surface irregularity at an acquisition speed of 10 mm^2^/10 s with 1.1‐µm lateral resolution (Figure S9, Supporting Information), leading to a high imaging throughput of ∼200 megapixels (throughput is defined by the ratio of attainable FOV per minute to the square of the half‐pitch resolution, thus the throughput of CHAMP is calculated as 60 mm^2^ / (1.1 µm/2)^2^ ≈ 200 megapixels. The throughput across different imaging modalities is compared in Figure S2, Supporting Information).

The spatial resolution of CHAMP was measured by imaging 500‐nm‐diameter fluorescent beads (B500, excitation/emission: 365/445 nm, Thermo Fisher Scientific Inc.) (Figure S9, Supporting Information). The Gaussian‐fitted data show that the full width at half maximum is 1.1 µm in the reconstructed CHAMP image while 2.9 µm in the diffraction‐limited wide‐field image, demonstrating 2.6× resolution enhancement through speckle illumination.

### Virtual Staining through Unsupervised Learning

Figure S10, Supporting Information shows the architecture of the generator and discriminator networks. The objective of CycleGAN contains two types of loss functions — adversarial loss^[^ ^62^ ^]^ and cycle consistency loss. ^[^ ^31^ ^]^ For adversarial loss, the objective of the discriminator *Y* (*D*_Y_) is calculated as:

(1) $$\begin{array}{ccc} L_{GAN}\;\left(G,D_{Y},X,Y\right) & = & E_{y\to p_{data\left(y\right)}}\;\left[\log D_{Y}\left(y\right)\right]\\ & & +E_{x\to p_{data\left(x\right)}}\left[\log\left(1-D_{Y}\left(G\left(x\right)\right)\right)\right] \end{array}$$

Similarly, the objective of the discriminator X (*D_X_*) is:

(2) $$\begin{array}{ccc} L_{GAN}\;\left(F,D_{X},X,Y\right) & = & E_{x\to p_{data\left(x\right)}}\;\left[\log D_{X}\left(x\right)\right]\\ & & +E_{y\to p_{data\left(y\right)}}\left[\log\left(1-D_{X}\left(F\left(y\right)\right)\right)\right] \end{array}$$

Cycle consistency loss, which is applied to monitor the training process, is calculated as:

(3) $$\begin{array}{ccc} L_{cyc}\;\left(G,F\right) & = & E_{x\to p_{data\left(x\right)}}\;\left[∥F\left(G\left(x\right)\right)-x∥\right]\\ & & +E_{y\to p_{data\left(y\right)}}\left[∥G\left(F\left(y\right)\right)-y∥\right] \end{array}$$

In addition, structural similarity index measure (SSIM),^[^ ^63^ ^]^ which predicts the perceived quality based on illuminance, contrast, and structure, is appended to the aforementioned loss functions. The SSIM loss is calculated as:

(4) $$\begin{array}{ccc} L_{ssim}\;\left(G,F\right) & = & \left(1-E_{x\to p_{data\left(x\right)}}\left[\mathrm{SSIM}\left(x,G\left(x\right)\right)\right]\right)\;\\ & & +\left(1-E_{y\to p_{data\left(y\right)}}\left[\mathrm{SSIM}\left(y,F\left(y\right)\right)\right]\right) \end{array}$$

The overall objective for our virtual staining network is the weighted sum of the four loss functions, which is given by:

(5) $$\begin{array}{ccc} l\;\left(G,\;F,\;D_{X},\;D_{Y}\right) & = & L_{GAN}\;\left(G,D_{Y},X,Y\right)+L_{GAN}\left(F,D_{X},X,Y\right)\\ & & +\lambda L_{cyc}\left(G,F\right)+\gamma L_{ssim}\left(G,\;F\right) \end{array}$$

where *λ* is set to 10 and *γ* is set to 2. The network is implemented with Python version 3.7.3 and Pytorch version 1.0.1. The software is implemented on a desktop computer with a Core i7‐9700K CPU@ 3.6GHz and 64GB RAM, running on an Ubuntu 18.04.2 LTS operation system. The training and testing are performed by an NVIDIA Titan RTX GPU with 24 GB RAM, which allows operating on ∼25 megapixels/s for testing (including GPU computing and time to write to hard disk).

For virtual staining network of fixed mouse brains, the training data consists of 1600 unpaired CHAMP and H&E‐stained images, where CHAMP images were collectively obtained from fixed, thick/thin mouse brains, and histological images were collected from H&E‐stained thin mouse brain slices which contain similar features as the CHAMP images. For the virtual staining network of fresh mouse brains, the training data consists of 800 unpaired CHAMP and H&E‐stained images, where CHAMP images were collectively obtained from freshly excised mouse brains. For the style transformation network, the training data consists of 800 unpaired “brain‐style” Deep‐CHAMP images and H&E‐stained images of thin mouse kidney slices, where Deep‐CHAMP images were the output from the fixed mouse brain network with the CHAMP images of mouse kidney used as the input. The training details and convergence plots can be found in Figure S11, Supporting Information.

To show the wide applicability of the unsupervised network, the fixed mouse brain/kidney tissues with various thicknesses were utilized for cross validation. The resulting virtually stained Deep‐CHAMP images are enumerated in Figure S12, Supporting Information. It is emphasized that the CycleGAN‐based network enables image translation without paired training data, thus fundamentally favoring the virtual staining of CHAMP images of thick and unprocessed tissues. The network was trained with the hybrid CHAMP images of unprocessed/processed, thick/thin tissues that can exhibit differences in terms of cellular morphology, image contrast, and brightness, thus demonstrating strong applicability in different tissue thicknesses (Figure S12i,j, Supporting Information). In addition, the bridge network enables style transformation from “brain‐style” Deep‐CHAMP images (Figure S12k,l, Supporting Information) to “kidney‐style” Deep‐CHAMP images (Figure S12m,n, Supporting Information) without the need for retraining a kidney network. Because of this, cross‐organ validation with CycleGAN is feasible. This transformation is also applicable to other different types of tissues as long as the CHAMP images of these tissues do not show a significant difference with the mouse brain, showing the great simplicity and flexibility of the unsupervised neural network.

### Calculations of Cross‐Sectional Area and Intercellular Distance and Statistical Analysis

Deep‐CHAMP and H&E‐stained histological images were segmented by a free Fiji plugin, trainable Weka segmentation,^[^ ^64^ ^]^ which enables to produce pixel‐based segmentations. Based on the resulting probability maps, images were subsequently converted to a binary image where cell nuclei can be identified. The binarized Deep‐CHAMP and H&E‐stained images were analyzed in Fiji, where the cross‐sectional area and centroid of each nucleus were provided. With the localized center positions of the cell nuclei, the intercellular distance was calculated to be the shortest adjacent distance to a neighboring cell nucleus. A two‐sided Wilcoxon rank sum test was carried out across groups with *n* = 50 for each distribution of cross‐sectional area and intercellular distance. No assumptions were made on data distributions. The significance was defined as p* ≤ 0.05 in all cases. Statistical analysis was carried out using MATLAB (MATLAB R2018b, MathWorks, Inc).

## Conflict of Interest

T.T.W.W. has a financial interest in PhoMedics Limited, which, however, did not support this work. Y.Z., L.K., I.H.M.W., X. L., and T.T.W.W. have applied for a patent (US Provisional Patent Application No.: 62/973 101) related to the work reported in this manuscript.

## Author Contributions

Y.Z. and L.K. contributed equally to the work. Y.Z., L.K., and T.T.W.W. conceived of the study. Y.Z. and L.K. built the imaging system. Y.Z., L.K., X.L., and M.K.Y.H. prepared the specimens involved in this study. Y.Z. and I.H.M.W. performed imaging experiments. L.K. and W.D. performed histological staining. Y.Z. processed the data. Y.Z. and R.C.K.C. analyzed the data. Y.Z. and T.T.W.W. wrote the manuscript. T.T.W.W. supervised the whole study.

## Supporting information

Supporting Information

Supplemental Video 1

Supplemental Video 2

Supplemental Video 3

Supplemental Video 4

## Data Availability

The data that support the findings of this study are available from the corresponding author upon reasonable request. The customized code in MATLAB for CHAMP super‐resolution reconstruction is available at https://github.com/TABLAB‐HKUST/CHAMP. The code of virtual staining networks based on the architecture of CycleGAN is available at https://github.com/TABLAB‐HKUST/Deep_CHAMP.
