# Supplementary material for: High‐Throughput, Label‐Free and Slide‐Free Histological Imaging by Computational Microscopy and Unsupervised Learning
Source: Adv Sci (Weinh). 2021 Nov 7;9(2):2102358. doi: 10.1002/advs.202102358 (PMC8805566; doi:10.1002/advs.202102358)
Supplement: Supplementary file 1 — Supporting Information [file ADVS-9-2102358-s005.pdf]

ADVANCED SCIENCE
Open Access

Supporting Information

for *Adv. Sci.*, DOI: 10.1002/advs.202102358

High-throughput, label-free and slide-free histological imaging by computational microscopy and unsupervised learning

*Yan Zhang, Lei Kang, Ivy H. M. Wong, Weixing Dai, Xiufeng Li, Ronald C. K. Chan, Michael K. Y. Hsin, Terence T. W. Wong**

High-throughput, label-free and slide-free histological imaging by computational microscopy and unsupervised learning

Yan Zhang[1,#], Lei Kang[1,#], Ivy H. M. Wong[1], Weixing Dai[1], Xiufeng Li[1], Ronald C. K. Chan[2], Michael K. Y. Hsin[3], Terence T. W. Wong[1,]*

[1]*Translational and Advanced Bioimaging Laboratory, Department of Chemical and Biological Engineering, The Hong Kong University of Science and Technology, Kowloon, Hong Kong, China*

[2]*Department of Anatomical and Cellular Pathology, The Chinese University of Hong Kong, Shatin, Hong Kong, China*

[3]*Department of Cardiothoracic Surgery, Queen Mary Hospital, Hong Kong, China*

[#]*These authors contributed equally to the work*

[*]*Corresponding author: ttwwong@ust.hk*

The Supplementary Material includes:

Figure S1 | Workflow of surgical margin assessment.

Figure S2 | Comparison of state-of-the-art slide-free imaging modalities.

Figure S3 | Contrast difference of a mouse spleen with excitation wavelengths of 265 nm and 340 nm.

Figure S4 | CHAMP and Deep-CHAMP validation with thin mouse brain/kidney slices.

Figure S5 | CHAMP and Deep-CHAMP validation with a thin human lung cancer tissue slice.

Figure S6 | CHAMP and Deep-CHAMP validation with fixed and unprocessed mouse brain/kidney tissues.

Figure S7 | CHAMP and Deep-CHAMP validation with a freshly excised mouse kidney tissue.

Figure S8 | Illustration of intensity modulation by structured illumination microscopy.

Figure S9 | Experimental characterization of CHAMP's lateral resolution.

Figure S10 | Architecture of generator and discriminator neural networks.

Figure S11 | Convergence plots and training details.

Figure S12 | Cross-validation of the virtual staining network.

Table S1 | Flowchart of super-resolution reconstruction framework.

Other Supplementary Material for this manuscript includes the following:

Video S1 | System setup, image acquisition, and data processing.

Video S2 | A series of close-up and registered CHAMP, Deep-CHAMP, and H&E-stained histological images of a thin mouse brain slice.

Video S3 | A series of close-up and registered CHAMP, Deep-CHAMP, and H&E-stained histological images of formalin-fixed thick mouse brain tissues.

Video S4 | A series of close-up and registered CHAMP and Deep-CHAMP images of vibratome-cut mouse brain/kidney tissues.

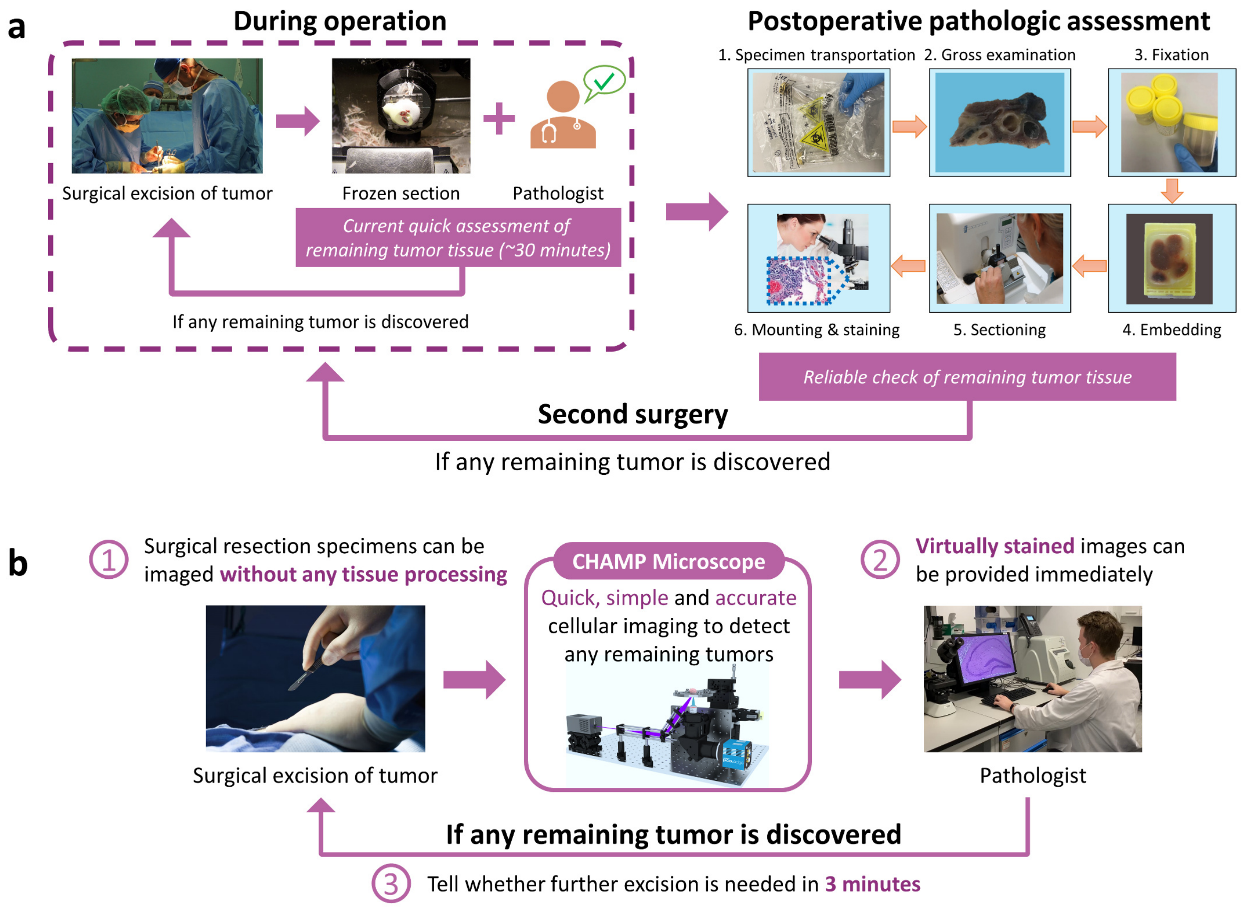

Figure S1. Workflow of surgical margin assessment. a, Conventional standard-of-care histopathology, which involves two approaches, including (1) intraoperative frozen section with freshly excised tissues, and (2) postoperative assessment with formalin-fixed and paraffin-embedded tissues. **b**, Anticipated new clinical practice by using the CHAMP microscope.

Method	Image acquisition	Contrast mechanism	Optical sectioning ability	Imaging throughput	Ease of use	Cost-effectiveness
LSCM	2D/3D laser scanning	Exogenous/endogenous fluorescence	★★★★<br>Controlled by pinhole size, can be traded with SNR and signal intensity	★★<br>~10 megapixels<br>Determined by laser scanning speed and dwell time	★★<br>High system complexity in terms of optical alignment, system synchronization, and maintenance	★★<br>Require high-speed laser scanning module
MPM			★★★★★<br>Controlled by the size of tightly-focused beam	★<br>~2 megapixels @ 80 MHz laser<br>Determined by laser repetition rate and dwell time		★<br>Require ultrafast pulsed laser with high peak power, and high-speed laser scanning module
SRS		Intrinsic vibration of lipids, proteins, and nucleic acids				
SHG		Non-centrosymmetric properties of endogenously orientated structure				
PAM		Optical absorption of endogenous biomolecules	★★<br>Controlled by the bandwidth of ultrasonic transducer	★★<br>~10 megapixels @ 100 kHz laser<br>Determined by laser repetition rate and dwell time	★★★<br>Require coupling media and no image can be provided in real time	★★<br>Require pulsed laser with high energy, and high-speed laser scanning module
LSFM	Wide-field illumination	Two exogenous fluorescence analog of H&E	★★★★<br>Controlled by the thickness of light-sheet, can be traded with field-of-view	★★★★★<br>~850 megapixels<br>Determined by exposure time and light sheet thickness	★★★<br>Tissue clearing for volumetric imaging<br>★★★<br>Require well-regulated staining protocol to avoid fluorescence saturation and leakage	★★★<br>Require laser and high-speed camera
SIM			★★★<br>Controlled by the frequency of illumination pattern, can be traded with SNR and imaging depth	★★★★★<br>~800 megapixels<br>Determined by exposure time and pattern switching time	★★★<br>Precisely controlled pattern generation module	★★★★<br>Require spatial light modulator and camera with high dynamic range
MUSE			★★<br>Controlled by the UV penetration depth, which is tissue dependent	★★★★<br>~110 megapixels<br>Determined by exposure time and number of axial scanning for extended DOF	★★★★<br>Variable focusing is required for surface irregularities	★★★★★<br>Only require UV LED
CHAMP		Endogenous fluorescence		★★★★<br>~200 megapixels<br>Determined by exposure time and number of acquisitions required for super-resolution reconstruction	★★★★★<br>Label-free, large DOF accommodates tissue irregularities	★★★★<br>Require UV laser

Figure S2. Comparison of state-of-the-art slide-free imaging modalities. The throughput of each imaging modality is calculated based on the reported literature, including laser scanning confocal microscopy (LSCM)[1], multiphoton microscopy (MPM)[2], stimulated Raman scattering (SRS) and second harmonic generation (SHG)[3], photoacoustic microscopy (PAM)[4], light-sheet fluorescence microscopy (LSFM)[5], structured illumination microscopy (SIM)[6], and microscopy with ultraviolet surface excitation (MUSE)[7].

* Note. Throughput is defined by the ratio of attainable field-of-view per minute to the square of half-pitch resolution. For an easy comparison, computational time and fluorescence labeling time are not considered here. Throughput is calculated for each imaging modality under the same level of tissue irregularity of 80 µm. For instance, in LSCM[1], 10 mm^2/minute with 0.6-µm lateral resolution and 8-µm focus tracking is achieved, such that the throughput is calculated as $\frac{10\ \mathrm{mm}^2/(0.6\ \mu\mathrm{m}/2)^2}{(80\ \mu\mathrm{m}/8\ \mu\mathrm{m})} \approx 10$ megapixels. In nonlinear microscopy, including MPM[2], SRS, and SHG[3], 1 mm^2/minute with 0.4-µm lateral resolution is obtained, and the axial scanning interval is 5 µm for each slice, such that the throughput is calculated as $\frac{1\ \mathrm{mm}^2/(0.4\ \mu\mathrm{m}/2)^2}{(80\ \mu\mathrm{m}/5\ \mu\mathrm{m})} \approx 2$ megapixels. In LSFM[5], 100 mm^2/12.5 s at 80-µm depth-of-field (DOF) with 1.5-µm lateral resolution is achieved, such that the throughput is calculated as $480\ \mathrm{mm}^2/(1.5\ \mu\mathrm{m}/2)^2 \approx 850$ megapixels. In MUSE[7], 110 mm^2/minute with 0.7-µm lateral resolution is achieved, and multiple z-stacks are acquired at 10-µm spacing for extended DOF, such that the throughput is calculated as $\frac{110\ \mathrm{mm}^2/(0.7\ \mu\mathrm{m}/2)^2}{(80\ \mu\mathrm{m}/10\ \mu\mathrm{m})} \approx 110$ megapixels. In CHAMP, 60 mm^2/minute with 1.1-µm lateral resolution at 80-µm DOF is achieved, such that the throughput is calculated as $60\ \mathrm{mm}^2/(1.1\ \mu\mathrm{m}/2)^2 \approx 200$ megapixels.

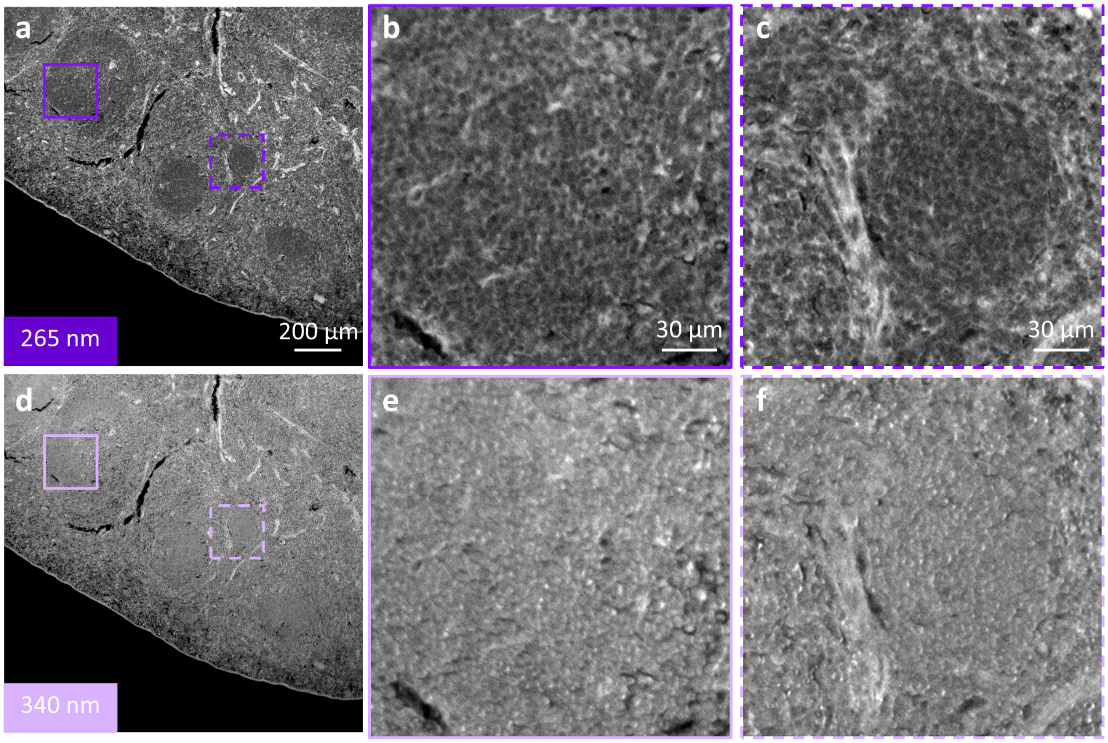

Figure S3. Contrast difference of a mouse spleen with excitation wavelengths of 265 nm and 340 nm. **a,** CHAMP image of a thin mouse spleen slice excited by a 265-nm light-emitting-diode (LED). **b,c,** Zoomed-in images of solid and dashed regions in a, respectively. **d,** CHAMP image of the same thin mouse spleen slice excited by a 340-nm LED. **e,f,** Zoomed-in images of solid and dashed regions in d, respectively.

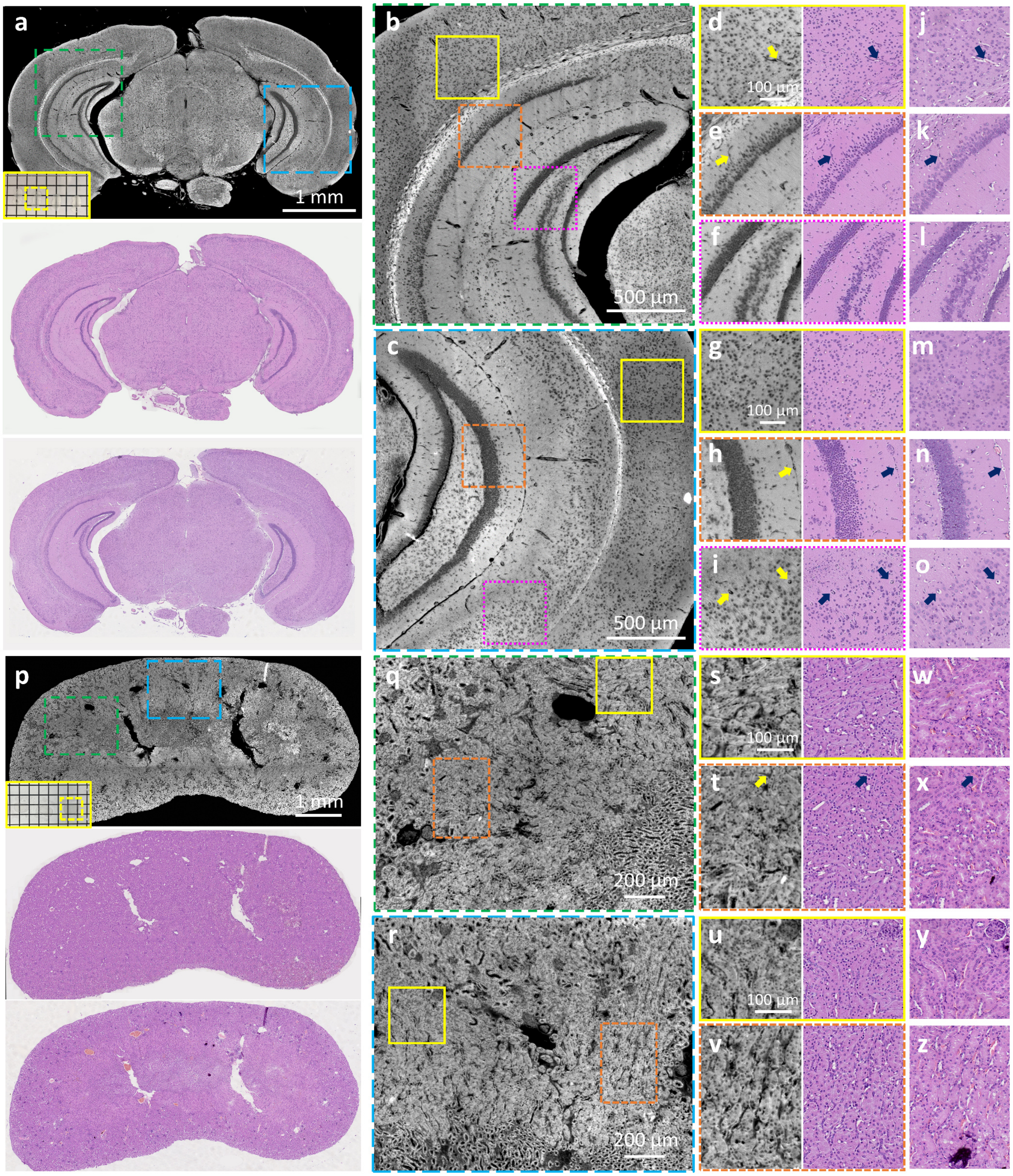

Figure S4. CHAMP and Deep-CHAMP validation with thin mouse brain/kidney slices. a, CHAMP (top), Deep-CHAMP (middle), and H&E-stained image (bottom) of a thin mouse brain slice, inset at the bottom left of CHAMP shows the photograph of the specimen (the yellow dashed box shows the slice that is imaged). **b**,**c**, Zoomed-in CHAMP images of green and blue dashed regions in a, respectively. **d**–**f**,**g**–**i**, Zoomed-in CHAMP and Deep-CHAMP images of yellow solid, orange dashed, and magenta dashed regions in b and c, respectively. **j**–**o**, The corresponding H&E-stained histological images. **p**, CHAMP (top), Deep-CHAMP (middle), and H&E-stained image (bottom) of a thin mouse kidney slice, inset at the bottom

left of CHAMP shows the photograph of the specimen (the yellow dashed box shows the slice that is imaged). **q**,**r**, Zoomed-in CHAMP images of green and blue dashed regions in p, respectively. **s**–**v**, Zoomed-in CHAMP and Deep-CHAMP images of yellow solid and orange dashed regions in q and r, respectively. **w**–**z**, The corresponding H&E-stained histological images. Arrows indicate the segmentation-induced staining artifacts in Deep-CHAMP images.

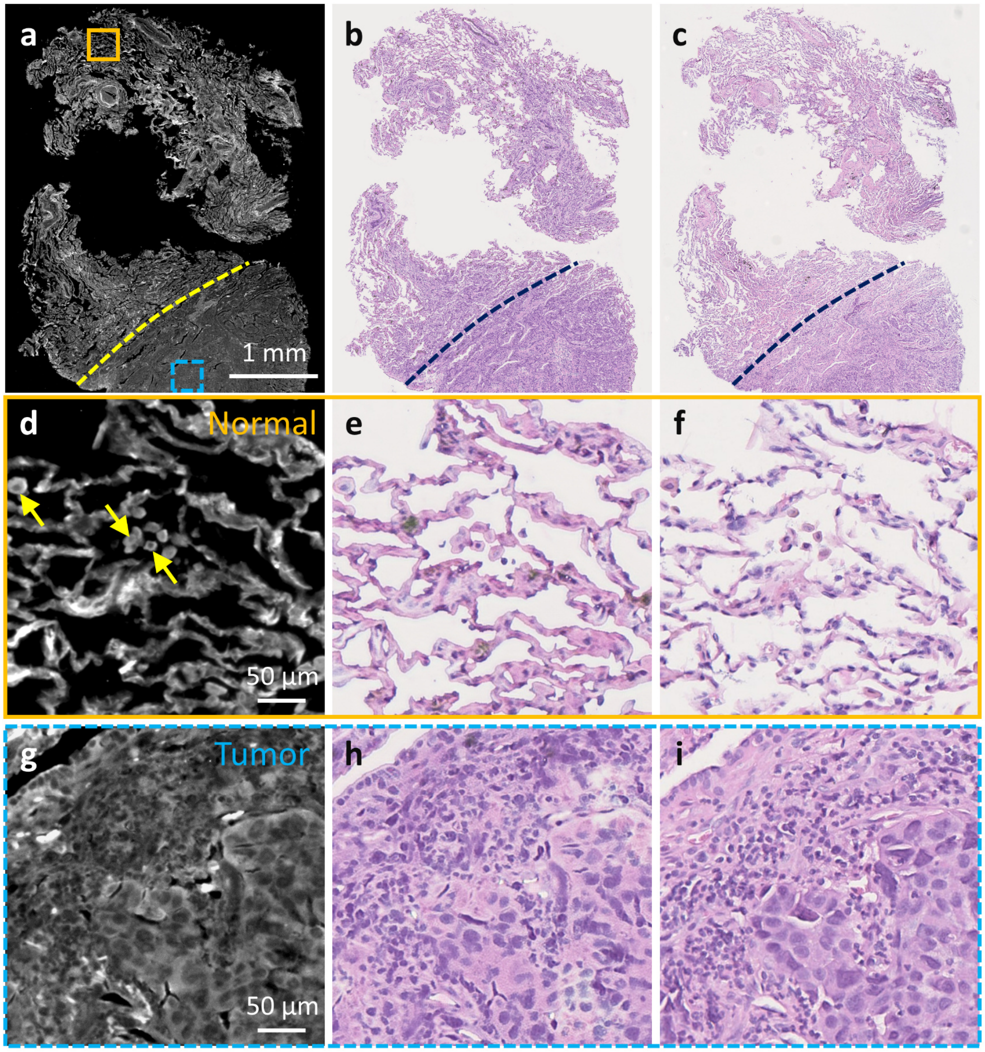

Figure S5. CHAMP and Deep-CHAMP validation with a thin human lung cancer tissue slice. a–c, CHAMP, Deep-CHAMP, and H&E-stained histological images of a thin human lung cancer tissue slice with large cell carcinoma, respectively. The dashed curves outline the interface between normal and tumor regions. **d–f, g–i,** CHAMP, Deep-CHAMP, and H&E-stained histological images of orange solid and blue dashed regions in a, respectively. Arrows indicate the alveolar macrophages.

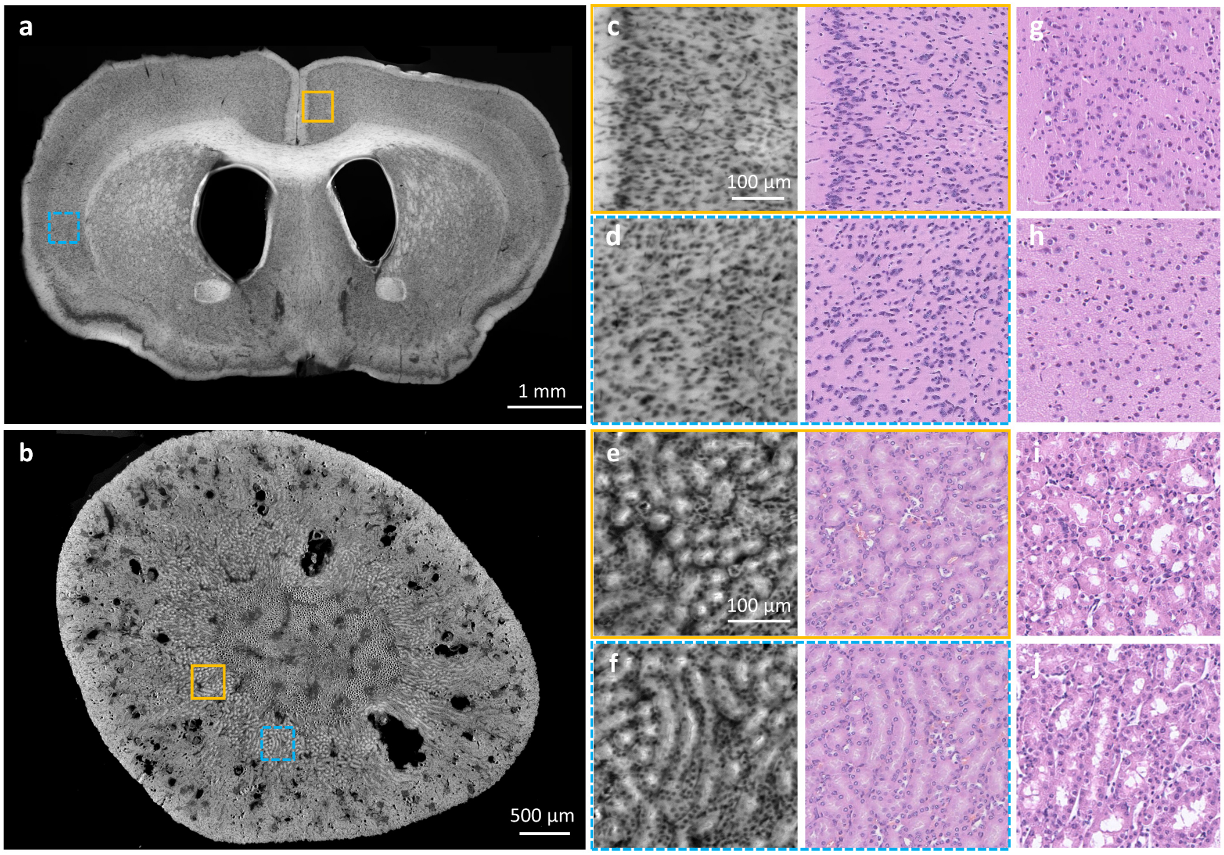

Figure S6. CHAMP and Deep-CHAMP validation with fixed and unprocessed mouse brain/kidney tissues. a,b, CHAMP images of formalin-fixed and unprocessed mouse brain and kidney tissues (~200-µm thickness), respectively. **c–f**, Zoomed-in CHAMP and Deep-CHAMP images of orange solid and blue dashed regions in a and b, respectively. **g–j**, The corresponding H&E-stained histological images.

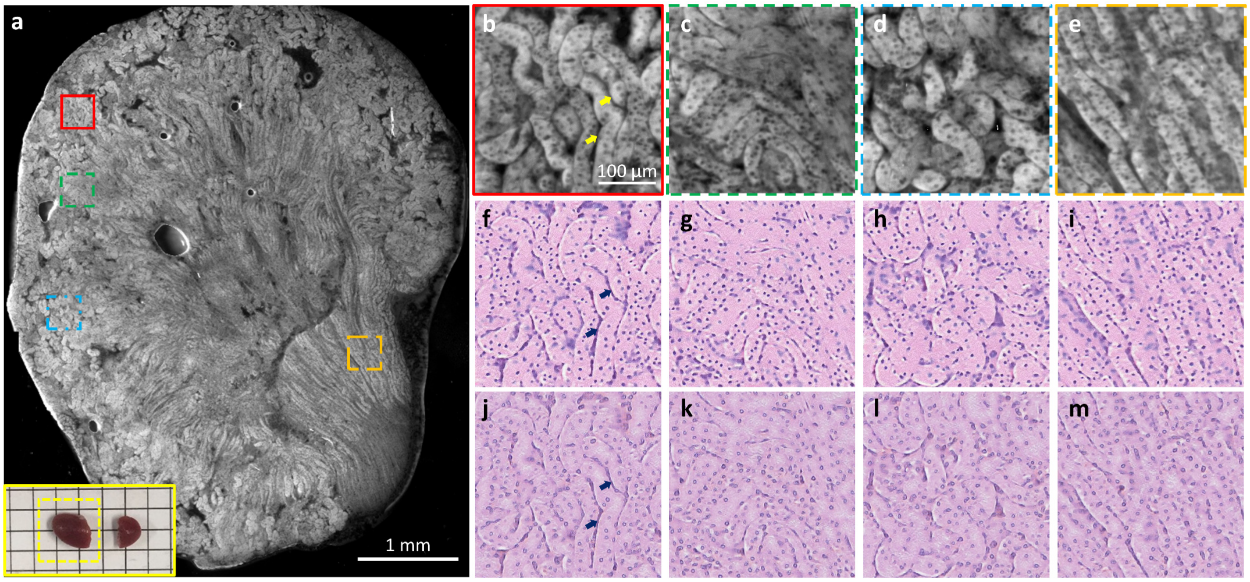

Figure S7. CHAMP and Deep-CHAMP validation with a freshly excised mouse kidney tissue. a, CHAMP image of a freshly excised mouse kidney tissue, inset at the bottom left shows the photograph of the specimen (the yellow dashed box shows the mouse kidney that is imaged). **b**–**e**, Zoomed-in CHAMP images of red solid, green dashed, blue dashed, and orange dashed regions in a, respectively. **f**–**i**, The corresponding 'brain-style' Deep-CHAMP images output by the virtual staining network trained for mouse brain. **j**–**m**, The corresponding 'kidney-style' Deep-CHAMP images output by the style transformation network with f–i as the input. Arrows indicate the segmentation-induced staining artifacts in Deep-CHAMP images.

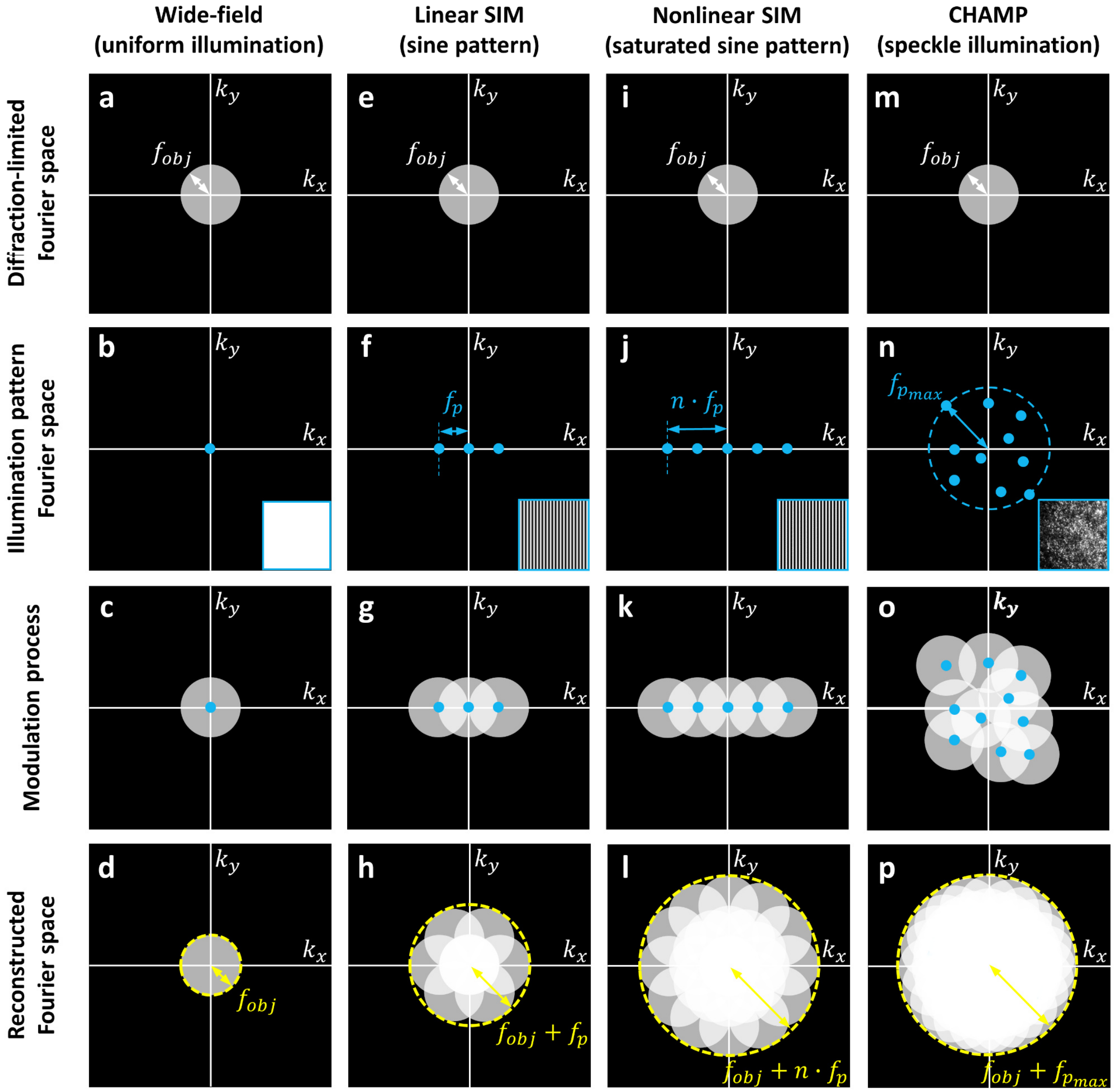

Figure S8. Illustration of intensity modulation by structured illumination microscopy. a–d, Diffraction-limited wide-field imaging with uniform illumination. **e–h**, Linear SIM with sinusoidal illumination. **i–l**, Nonlinear SIM with saturated sinusoidal illumination. **m–p**, CHAMP imaging with translated speckle illumination. For SIM, the sinusoidal pattern is phase-shifted and rotated to synthesize an isotropic aperture (h,l). While for CHAMP, the speckle pattern is translated to isotropically fill the Fourier space (p). f_{obj}: frequency of objective lens, f_p: frequency of sinusoidal pattern, n: the order of sinusoidal harmonics, $f_{p_{max}}$: the maximum frequency of the speckle pattern.

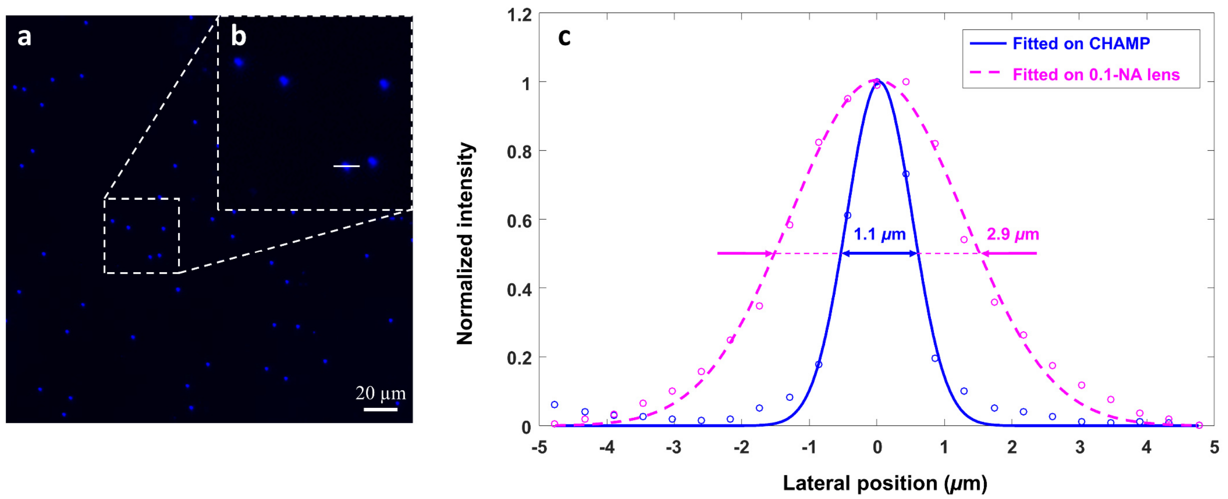

Figure S9. Experimental characterization of CHAMP's lateral resolution. a, CHAMP image of blue fluorescent beads (500-nm in diameter with an emission wavelength of 445 nm). **b**, Zoomed-in CHAMP image of the white dashed box in a. **c**, Gaussian-fitted intensity distribution along the solid line in b, showing that the full width at half maximum is 1.1 µm in CHAMP (blue solid line) and 2.9 µm in wide-field microscopy with a 0.1-NA objective lens (magenta dashed line).

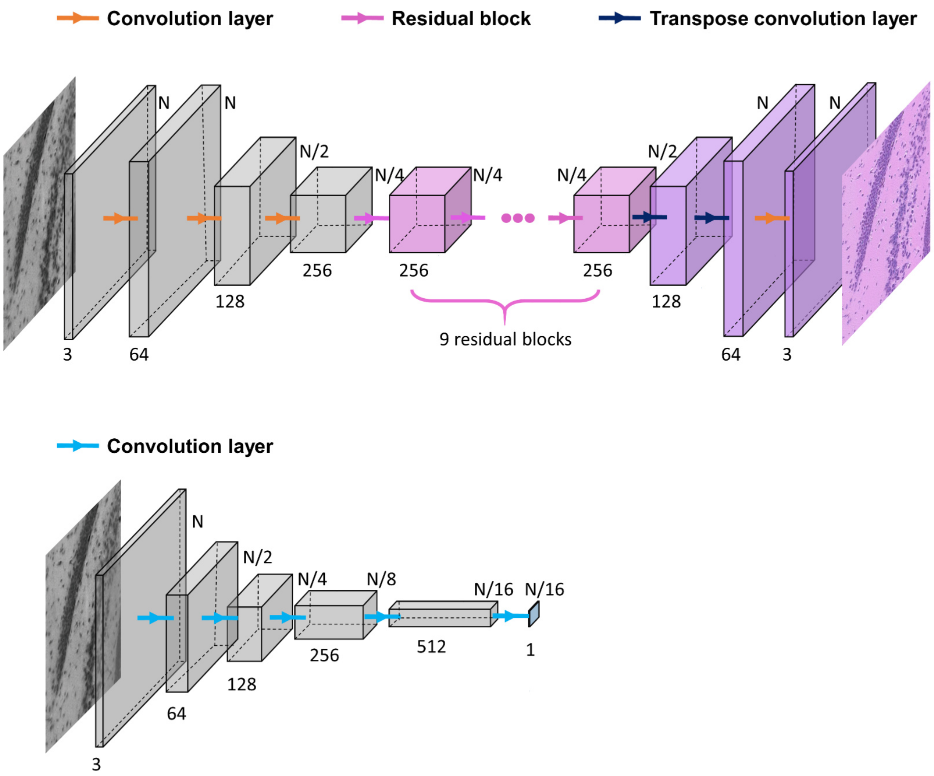

Figure S10. Architecture of generator and discriminator neural networks. The Resnet-based generator network[8] (top) consists of a downsampling path (gray), a residual path (pink), and an upsampling path (purple). The first convolution layer (kernel 7 × 7, stride 1 × 1) in the downsampling path increases the image channel to 64 with size unchanged, while the other two layers (kernel 3 × 3, stride 2 × 2) will halve the image size and double the image channel. The network is then followed by 9 residual blocks, in which the image size and channel will remain unchanged. In the upsampling path, the first two layers (kernel 3 × 3, stride 2 × 2) will double the image size and halve the image channel, while the third layer (kernel 7 × 7, stride 1 × 1) decreases the image channel to 3. The last convolution layer is followed by a hyperbolic tangent activation while other convolution layers are followed by an instance normalization and rectified linear unit (ReLU) activation. The PatchGAN-based discriminator network[9] (below) consists of 5 convolutional layers. The image size will be halved by each of the first 4 convolution layers (kernel 4 × 4, stride 2 × 2), with each layer followed by an instance normalization and Leaky-ReLU activation. The last layer (kernel 4 × 4, stride 1 × 1) decreases the channel to 1 to output the probability labels.

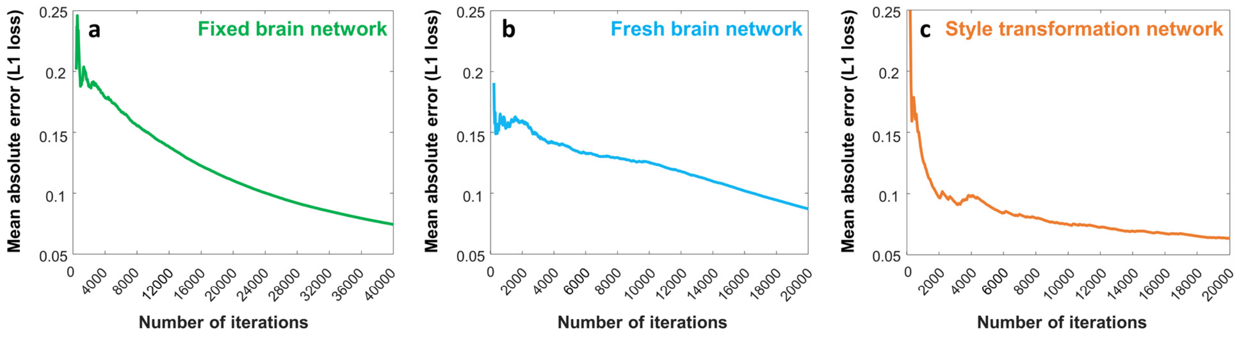

Network	# of epochs	# of iterations	# of patches (256 × 256 pixels)	Batch size	Training time (h)
Fixed brain network	100	40000	1600	4	8
Fresh brain network	100	20000	800	4	4
Style transformation network	100	20000	800	4	4

Figure S11. Convergence plots and training details. a–c, L1-loss (moving average) with respect to the number of iterations of the fixed brain network, fresh brain network, and style transformation network, respectively. The table below shows the parameters of the neural networks a–c.

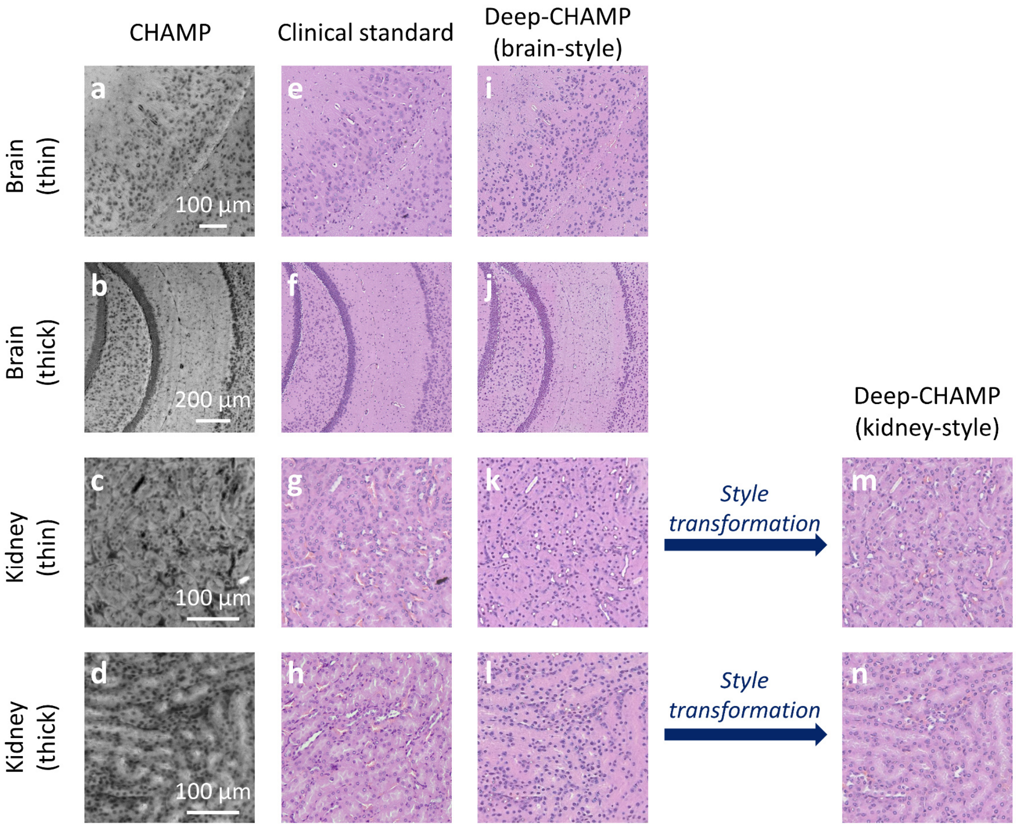

Figure S12. Cross-validation of the virtual staining network. a–d, CHAMP images of formalin-fixed mouse brain/kidney tissues with varying thickness. **e–h**, The corresponding H&E-stained images. **i–l**, The corresponding 'brain-style' Deep-CHAMP images output by the virtual staining network for fixed mouse brains. **m**,**n**, The corresponding 'kidney-style' Deep-CHAMP images output by the style transformation network with k and l as the input, respectively.

Reconstruction Framework

Input: 36 speckle-illuminated autofluorescence images I_j and pre-estimated position shifts (x_j, y_j)
Output: Resolution-enhanced object $o(x, y)$ and unknown speckle pattern $p(x, y)$

**1.**	Initialize $o(x,y)$ and $p(x,y)$
**2.**	for $\text{iteration} = 1{:}\,10$
**3.**	for $j = 1{:}\,36$
**4.**	$o_j = o(x - x_j, y - y_j)$
**5.**	$\varphi_j(x,y) = o_j \cdot p(x,y)$
**6.**	$\psi_j(k_x,k_y) = F\left(\varphi_j(x,y)\right) \cdot OTF(k_x,k_y)$
**7.**	$I_{est} = \left|F^{-1}\left(\psi_j(k_x,k_y)\right)\right|$
**8.**	$I_{est}^{update}(1{:}\,3{:}\,3\mathrm{M}, 1{:}\,3{:}\,3\mathrm{M}) = I_j$
**9.**	$\mathrm{F}(\varphi_j^{update}) = F(\varphi_j) + \frac{conj(OTF) \cdot \left[F\left(I_{est}^{update}\right) - \psi_j\right]}{(1-\alpha)\lvert OTF\rvert^2 + \alpha\lvert OTF\rvert_{max}^2}$
**10.**	$o_j = o_j + \frac{conj(p) \cdot \left(\varphi_j^{update} - \varphi_j\right)}{(1-\beta)\lvert p\rvert^2 + \beta\lvert p\rvert_{max}^2}$
**11.**	$p = p + \frac{conj(o_j) \cdot \left(\varphi_j^{update} - \varphi_j\right)}{(1-\gamma)\lvert o_j\rvert^2 + \gamma\lvert o_j\rvert_{max}^2}$
**12.**	$o(x,y) = o_j(x + x_j, y + y_j)$
**13.**	end
**14.**	end

Table S1. Flowchart of super-resolution reconstruction framework. The object $o(x, y)$ is firstly initialized by averaging all captured raw images which are correspondingly back-shifted according to the pre-estimated scanning trajectory (x_j, y_j), and followed by zero-padding in the Fourier domain from the size of M × M to 3M × 3M. Similarly, the speckle pattern $p(x, y)$ is initialized by averaging all captured raw images and padded in the Fourier domain to a size of 3M × 3M. For the jth captured image, the pattern $p(x, y)$ is multiplied with a shifted object $o(x - x_j, y - y_j)$ and Fourier transformed into the frequency domain, which is subsequently lowpass filtered by the optical transfer function of the imaging system, and inversely Fourier transformed to obtain an estimated output intensity I_{est}. This estimated intensity is updated by the correspondingly captured autofluorescence intensity I_j in the spatial domain with a sub-sampled method to bypass the resolution limit set by the physical pixel size. After that, the object o_j and speckle pattern p are alternately updated with the momentum-assisted regularized ptychographic iterative engine[10]. We adopt $\alpha = 1$ while $\beta = \gamma = 0.3$ in the updating function. The shifting operation (line 4 and line 12) is achieved by applying the angular spectrum in the frequency domain.

References

[1] J. Kang, I. Song, H. Kim, H. Kim, S. Lee, Y. Choi, H. J. Chang, D. K. Sohn, H. Yoo, *Quant. Imaging Med. Surg.* **2018**, *8*, 884.

[2] E. Olson, M. J. Levene, R. Torres, *Biomed. Opt. Express* **2016**, *7*, 3089.

[3] B. Sarri, R. Canonge, X. Audier, E. Simon, J. Wojak, F. Caillol, C. Cador, D. Marguet, F. Poizat, M. Giovannini, H. Rigneault, *Sci. Rep.* **2019**, *9*, 1.

[4] X. Li, L. Kang, Y. Zhang, T. T. W. Wong, *Opt. Lett.* **2020**, *45*, 5401.

[5] A. K. Glaser, N. P. Reder, Y. Chen, E. F. McCarty, C. Yin, L. Wei, Y. Wang, L. D. True, J. T. C. Liu, *Nat. Biomed. Eng.* **2017**, *1*, DOI: 10.1038/s41551-017-0084.

[6] T. C. Schlichenmeyer, M. Wang, K. N. Elfer, J. Q. Brown, *Biomed. Opt. Express* **2014**, *5*, 366.

[7] F. Fereidouni, Z. T. Harmany, M. Tian, A. Todd, J. A. Kintner, J. D. McPherson, A. D. Borowsky, J. Bishop, M. Lechpammer, S. G. Demos, R. Levenson, *Nat. Biomed. Eng.* **2017**, *1*, 957.

[8] J. Johnson, A. Alahi, L. Fei-Fei, in *Lect. Notes Comput. Sci. (Including Subser. Lect. Notes Artif. Intell. Lect. Notes Bioinformatics)*, Springer Verlag, **2016**, pp. 694–711.

[9] P. Isola, J.-Y. Zhu, T. Zhou, A. A. Efros, B. A. Research, *Image-to-Image Translation with Conditional Adversarial Networks*, **n.d.**

[10] A. Maiden, D. Johnson, P. Li, *Optica* **2017**, *4*, 736.
